# Targeted long-read methylation analysis using hybridization capture suitable for clinical specimens

**DOI:** 10.1016/j.crmeth.2025.101215

**Published:** 2025-11-03

**Authors:** Keisuke Kunigo, Satoi Nagasawa, Keiko Kajiya, Yoshitaka Sakamoto, Suzuko Zaha, Yuta Kuze, Akinori Kanai, Kotaro Nomura, Masahiro Tsuboi, Genichiro Ishii, Ai Motoyoshi, Koichiro Tsugawa, Motohiro Chosokabe, Junki Koike, Ayako Suzuki, Yutaka Suzuki, Masahide Seki

**Affiliations:** 1 Department of Computational Biology and Medical Sciences, Graduate School of Frontier Sciences, The University of Tokyo, 5-1-5 Kashiwanoha, Kashiwa, Chiba 277-8561, Japan; 2 Department of Thoracic Surgery, National Cancer Center Hospital East, 6-5-1 Kashiwanoha, Kashiwa, Chiba 277-8577, Japan; 3 Department of Pathology and Clinical Laboratories, National Cancer Center Hospital East, 6-5-1, Kashiwanoha, Kashiwa, Chiba 277-8577, Japan; 4 Department of Breast & Endocrine Surgery, St. Marianna University School of Medicine, 2-16-1, Sugao, Miyamae-ku, Kawasaki, Kanagawa 216-8511, Japan; 5 Department of Pathology, St. Marianna University School of Medicine, 2-16-1, Sugao, Miyamae-ku, Kawasaki, Kanagawa 216-8511, Japan; 6 Life Science Data Research Center, Graduate School of Frontier Sciences, The University of Tokyo, 5-1-5 Kashiwanoha, Kashiwa, Chiba 277-8561, Japan

**Keywords:** DNA methylation, target sequencing, long-read sequencing, phasing analysis, breast cancer, lung cancer, allele-specific methylation, nanopore sequencing

## Abstract

To detect precise DNA methylation patterns in long-read DNA sequencing analysis, an efficient target enrichment method is needed. In this study, we established t-nanoEM, a practical method that integrates a hybridization-based capture step into a long-read enzymatic methyl (EM)-seq library for nanopore sequencing. We achieved a high sequencing coverage of up to ×570 at 5 kb N50 in length. We applied this method to the long-read methylation analysis of cancers. Using breast cancer as an example, we demonstrated that the signature changes in DNA methylation occurring in local cell populations could be displayed in a haplotype-aware manner. In lung cancer, the spatial diversity in gene expression as detected by the spatial expression profiling analysis may be associated with changes in DNA methylation.

## Highlights

• T-nanoEM enables target region-specific long-read methylation analysis
• The method achieves high-depth methylation analysis with high sensitivity
• We provide a workflow for haplotype and mutated allele-specific methylation analysis
• We apply t-nanoEM to clinical breast and lung cancer tissue sections

## Motivation

Existing methods for targeted long-read methylation analysis, such as PCR, Cas9-based approaches, and adaptive sampling, are limited by the number of target sites and/or the amount of input DNA. While hybridization capture enables the analysis of numerous target genomic regions from small amounts of input DNA, its application to long-read methylation analysis has remained challenging. To address this limitation, we developed targeted nanoEM (t-nanoEM). T-nanoEM combines nanoEM, a method that enzymatically converts long DNA fragments to differentiate methylated and unmethylated cytosines for nanopore sequencing, with a hybridization capture system capable of capturing target DNA even after the conversion.

## Graphical abstract

**Figure:**
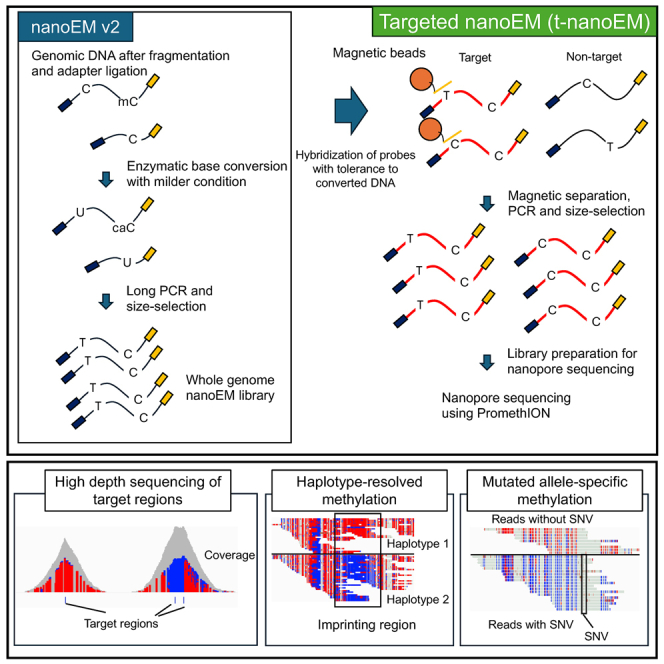

## Teaser

Kunigo et al. introduce t-nanoEM, a practical method that enables high-depth, target-specific long-read methylation analysis with as little as 8 ng of DNA. They develop a pipeline for haplotype-resolved and mutated allele-specific methylation analysis and demonstrate its utility by successfully applying it to clinical breast and lung cancer samples.

## Introduction

Nanopore sequencing of native genomic DNA (gDNA) can read over 10 kb in length. Base modifications, such as the methylation of cytosine on long DNA reads, can be detected from the electric patterns measured by nanopore sequencing.^1^^,^^2^ By combining these features, it is possible to analyze the DNA methylation pattern in long-read DNA, such as methylation analysis in a haplotype-aware manner.^3^^,^^4^^,^^5^ One drawback is that the required amount of DNA for this purpose is several hundred nanograms, which is not always feasible for the analysis of human disease specimens, such as most cancer specimens. To address this limitation, we previously developed a method known as nanoEM, which is a method for long-read methylation analysis starting from nanogram amounts of gDNA.^5^ In this method, an enzymatic base-conversion (EM) library was subjected to whole-genome nanopore sequencing. We demonstrated that nanoEM can be performed on a small amount of DNA (≥1 ng), and its N50 read length was retained at 5 kb. Despite the initial success, it was still difficult to precisely analyze methylation patterns in cancers, particularly those occurring in a small population of cancer cells. Thus, the sequencing depth obtained from a single flow cell was insufficient.

To improve upon the limited sequencing depth in nanopore sequencing, several methods have been developed (Figure S1A). The most direct approach is “adaptive sampling.”^6^^,^^7^^,^^8^ In this procedure, when the initial DNA bases do not match the preset target sequences, the DNA molecule is rejected from the nanopore and discarded. However, using this approach, the pores become inactive faster, resulting in a reduced total yield.^7^ Another approach for target enrichment of long-read sequencing is the selective retrieval of the target regions using Cas9.^9^ However, in this method, the number of target regions is limited to only several dozen because of the limitation of the number of guide RNAs. Adaptive sampling and Cas9 enrichment require several hundred nanograms and several micrograms of DNA as input, respectively. Therefore, a biochemical-based method for capture enrichment is needed to overcome these drawbacks.

For short-read methylation sequencing, several hybridization-based capture methods have been established and numerous applications have been reported in a high-throughput manner, even using small amounts of DNA.^10^^,^^11^ However, some modifications should be made when the hybridization capture method is used for the long-read methylation sequencing. First, hybridization-based capture methods generally require PCR amplification, which erases any trace of base modification; thus, the combination of base conversion, such as EM-seq, should be included in the procedure.^12^ Several methods for long-read methylation analysis have been described based on base conversion and target-specific long PCR^13^^,^^14^^,^^15^; however, it is difficult for PCR-based approaches to analyze a larger number of regions compared with hybridization capture-based approaches because they require primer design for each target region. Therefore, those methods involve amplicon sequencing and cannot be further scaled.

In this study, we developed targeted nanoEM (t-nanoEM), a method for long-read methylation analysis with high sequencing depth by combining hybridization capture and enzymatic base conversion. First, we optimized the procedure and evaluation of t-nanoEM using human breast cancer cell lines. We obtained reads with an N50 length of 5 kb and a high bait coverage (up to ×570). We constructed a pipeline for haplotype-resolved methylation analysis with the converted reads. Then, we applied t-nanoEM to samples prepared from clinical specimens.

## Results

### Development of a targeted capture method for long-read methylation sequencing (t-nanoEM)

**Figure 1:**
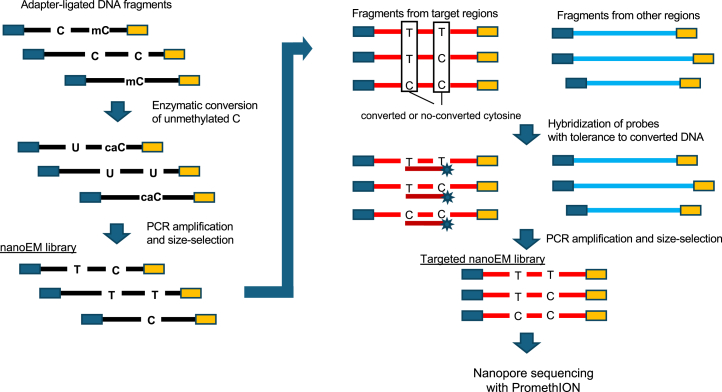
Schematic view of targeted nanoEM After adapter ligation to fragmented gDNA of 5–10 kb length, unmethylated cytosines on the DNA fragments were enzymatically converted to uridines. Using long PCR, the converted fragments were amplified by substituting the uridines with thymines. The amplified short fragments were removed with solid phase reversible immobilization (SPRI) beads. Using probes designed to capture DNA, even with base conversions, fragments derived from the target regions were selected from the nanoEM library. Using long PCR and subsequent size selection with SPRI beads, a t-nanoEM library was prepared. The t-nanoEM library was sequenced with the nanopore sequencer PromethION.

To expand the use of long-read DNA methylation sequencing starting from a small amount of DNA, we developed a method for targeted long-read methylation analysis by incorporating nanopore sequencing and EM-seq with the probe hybridization-based target enrichment method (designated as targeted nanoEM or t-nanoEM) (Figure 1). Although we also performed a simulation of adaptive sampling using nanoEM reads, the mapping rate on the CpG islands (CGIs) for the short sequences (150 bp) (such as those used to decide whether to continue sequencing or reject the DNA in adaptive sampling) was low (Figures S1B and S1C). Therefore, we did not incorporate adaptive sampling.

First, we modified the whole-genome nanoEM protocol, which is the material for target capture, to improve the complexity for deeper sequencing. A higher formamide concentration lowers the melting temperature of DNA^16^ and reduces the degradation of long DNA molecules. Therefore, we used a milder denaturation condition (66% formamide, at 80°C) before apolipoprotein B mRNA editing enzyme catalytic polypeptide (APOBEC) conversion compared with the standard protocol for EM-seq (20% formamide, at 85°C), which is also used for a similar method.^13^ Based on this modification, the yield of the nanoEM library was significantly improved (Figure S1D). To further evaluate the improved complexity of nanoEM (hereafter referred to as nanoEM v.2), we compared the sequencing data of the libraries between the original nanoEM (hereafter referred to as nanoEM v.1) and nanoEM v2, which were constructed from 50, 10, and 1 ng of input DNA extracted from the MDA-MB-231 (MB231) breast cancer cell line^5^ (Table S1A). As for the nanoEM v.2, the N50 lengths of the reads were improved, whereas the sequencing yields were slightly lower (for more details, see Figure S1E). The dataset for the nanoEM v.2 showed a significantly lower duplicate rate, particularly for small input amounts (Figure S1F). NanoEM v.2 showed a generally higher CpG coverage rate than v.1 (Figures S1G and S1H). Moreover, nanoEM v.2, even at 1 ng input, exhibited superior coverage compared with nanoEM v.1 at all input amounts. In fact, for nanoEM v.2, the correlation was the highest with the results of the short-read EM-seq (Figure S1I).

Using a nanoEM v.2 library as a starting material, we attempted to develop a method for long DNA fragment enrichment. After trying several alternative options (see Figures S2A–S2C for more details), we selected a base conversion aware capture (Twist Bioscience with some optimizations for long DNA: Figure 1; see STAR Methods for details). For this scheme, the base-converted and long PCR-amplified products were subjected to hybridization capture. For the probes, the converted sequence patterns were represented. More precisely, we modified the human methylome panel to capture long fragments targeting 123 Mb of the genomic regions, in which the probes were more sparsely tiled (Figure S2D). We examined and found that the Twist Standard Hyb and Wash Kit v.2 had superior performance compared with the Twist Fast Hybridization and Wash Kit, which are used for the short-read EM-seq target enrichment (Figure S2E; see also the next section). In addition, other reaction conditions, such as PCR and denaturation prior to hybridization of the probes, were modified (see Figures S2F and S2G for details).

**Figure 2:**
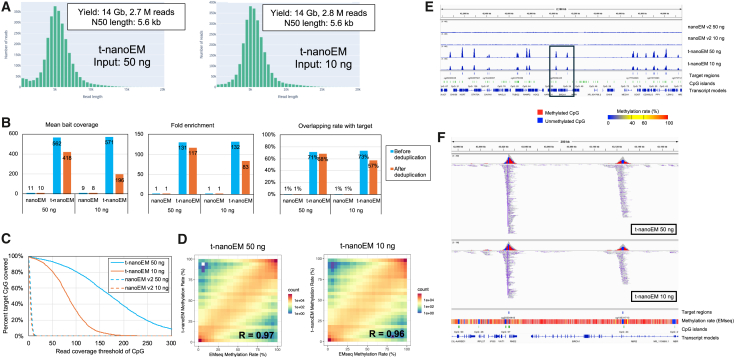
Assessment of t-nanoEM prepared with a pan-cancer panel The results of t-nanoEM prepared from 50 or 10 ng of MB231 gDNA with a pan-cancer panel. (A) Length distributions of 1d pass reads of t-nanoEM. Total sequenced bases (Gb), number, and N50 length of the t-nanoEM reads are shown in squares. (B) Mean bait coverage, fold enrichment, and overlapping rate before and after deduplication of t-nanoEM and nanoEM v.2 prepared from the same amount of gDNA with t-nanoEM are shown. (C) The percentage of CpG covered by the read coverage threshold of CpG by t-nanoEM and nanoEM v.2 after deduplication. (D) Scatterplots of the methylation rate of CpG covered by five reads or more between short-read EM-seq and t-nanoEM after deduplication. The frequency of dot counts within each bin is shown as a heatmap. (E and F) Typical views of the distribution of mapped reads after deduplication are displayed in the Integrative Genomics Viewer (IGV).^17^ Read coverage in nanoEM v.2 and t-nanoEM is shown in the top panel. The target regions, CGIs, and RefSeq transcript models are shown in the bottom panel. (F) Enlargement of the area enclosed by the square in (E). In the top panel, the read coverage and read distribution of t-nanoEM are shown by the bisulfite mode of IGV, in which methylated and unmethylated CpGs are shown in red and blue, respectively. Target regions, CGIs, and CpG methylation rate measured by short-read EM-seq,^5^ and transcript models are shown in the bottom panel. See also Tables S1 and S2.

After collecting the data, we determined whether the assembled protocol worked for t-nanoEM analysis. We constructed libraries starting with 10 ng of DNA extracted from two breast cancer cell lines (MB231 and BT474). We sequenced the libraries using a single flow cell of PromethION. We found that 3.7 and 3.9 M reads with read lengths of 6.3 and 5.5 kb could be successfully obtained (Figure S3A; Table S1A). We also determined whether the t-nanoEM library could be constructed using a panel smaller than the custom human methylome panel. We attempted to use a ready-made pan-cancer methylation panel targeting 1.5 Mb of genomics regions, which includes differentially methylated regions (DMR) in the The Cancer Genome Atlas (TCGA) database. For this pan-cancer panel, we successfully constructed the t-nanoEM libraries from 10 or 50 ng of input DNA of MB231, and 2.7 or 2.8 M reads at the N50 read length of 5.6 kb were obtained (Figure 2A; Table S1A).

### Evaluation of t-nanoEM

For the t-nanoEM data, we first compared the sequence profiles with those obtained from the short-read EM-seq data, which were obtained in our previous study.^5^ For the cancer panel, the mapping rate of t-nanoEM was higher (96%) compared with that of the whole-genome nanoEM v.2 (86%–88%) (Table S1A). Of these, >70% of the mapped reads overlapped the target regions (Figure 2B). The fold-enrichment and mean bait coverage scores were ∼130 and ∼570, respectively. Even after removing the PCR duplicates, high coverages were obtained. The mean bait coverage and overlapping rate for the target regions in nanoEM v.2 were ∼10% and 1%, respectively (Figure 2B). Even when focusing on the CpG sites, t-nanoEM showed higher coverage compared with nanoEM v.2 (Figure 2C). We also found that the correlation between the results of t-nanoEM and those of short-read EM-seq was very high (Figure 2D). These data collectively show that, with the t-nanoEM libraries, the DNA methylation of the target regions can be analyzed in a highly efficient and precise manner (Figures 2E and 2F).

Similar results were obtained from a larger panel, the custom human methylome panel (83 times larger than the pan-cancer panel). Overlapping rates of 95% and 94% were obtained for MB231 and BT474, respectively (Figure S3B). As a result, 17.4 and 18.1 of the sequencing coverage after deduplication were obtained from a single PromethION flow cell. The coverage of CpG sites and the correlation between the results of t-nanoEM and those of short-read EM-seq were also very high (Figures S3C and S3D). The results indicated that the t-nanoEM analysis can be used for further broad target regions. Interestingly, a trend was observed to show higher coverage in wide target regions compared with that in narrow regions probably because of the higher densities of the baits in those regions (Figure S3E). We also successfully confirmed the differential methylation of the promoter region of the PGR gene between MB231 and BT474 (Figure S3F), which is consistent with the results of previous studies.^5^^,^^18^ PGR is a molecular subtyping marker for breast cancer and is expressed in luminal-type breast cancers, including BT474, but suppressed in triple-negative types, such as MB231.^5^ The results suggested that t-nanoEM analysis is highly reproducible.

### Pooling analysis

To reduce cost, we considered pooling multiple samples before the capture step as well as demultiplexing the reads after sequencing. Therefore, nanoEM libraries with different unique dual index sequences (8 and 8 bp) were prepared from BT474 and MB231 DNA. The libraries were pooled and subjected to subsequent hybridization capture using the pan-cancer panel (Figure S3G). A total of 2.7 M reads at an average length of 5.7 kb were obtained (Table S1A). After trimming the P5 and P7 sequences outside of the indexes, the trimmed reads were demultiplexed using a custom script. As a result, 94% of the reads were assigned to either of the indexes (Figure S3H). To evaluate whether the reads were properly separated, we analyzed the assigned reads using our nanoEM analytical pipeline.^5^ We found that 95%–96% of the reads were aligned, and 74%–80% of the aligned reads overlapped the target regions (Table S1A; Figure S3I). Again, high fold-enrichment scores of 97 and 98 as well as the high mean bait coverage scores of 94 and 146 were observed, even after deduplication. We further compared the observed CpG methylation rate for each dataset with that obtained from the short-read EM-seq for the same cell line (Figure S3J). The CpG methylation rates of the demultiplexed nanoEM data exhibited high Pearson’s correlation coefficients. The trace of the mutual contamination of the reads from the other cell line was nearly absent (Figure S3K). The results indicate that the multiplexed data are comparable to single-plex data (Figure 2). Therefore, we concluded that the pooling analysis is possible for t-nanoEM analysis.

### Methylation analysis of the difficult regions for the short-read EM-seq

**Figure 3:**
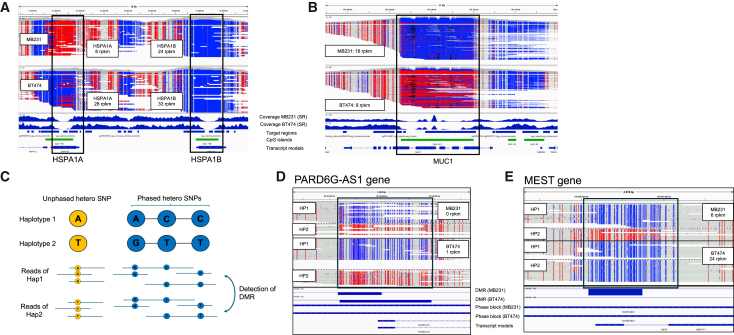
Methylation patterns of the difficult regions for short read and detection of haplotype-specific methylation status (A and B) Typical views of loci around HSPA1A and HSPA1B (A) and MUC1 (B), where short reads are hardly aligned. In the top panel, the coverage and distribution of t-nanoEM reads prepared from 10 ng of DNA from MB231 and BT474 with the human methylome panel after deduplication are shown. In the middle panel, the read coverages of the short-read EM-seq in MB231 and BT474^5^ are shown. The target regions, CGIs, and RefSeq transcript models are shown in the bottom panel. The expression (rpkm) of each gene is shown in each square. The RNA-seq expression data were obtained from the Cancer Cell Line Encyclopedia (CCLE).^24^ (C) Schematic view of the haplotype phasing of heterozygous SNPs and the detection of differentially methylated regions between haplotypes. (D and E) Examples of detected haplotype-specific DMRs around PARD6G-AS1 (D) and MEST genes (E). For each cell line, the reads for each haplotype and the CpG methylation statuses are visualized. DMRs between haplotypes and phased block, in which heterozygous SNPs are continuously phased, are shown in the middle panel. The transcript models are shown in the bottom panel. The expression of each gene is shown in each square. See also Tables S1 and S2 and Data S1.

Long-read sequencing can occasionally analyze regions that are difficult to cover with short reads, including repetitive regions.^19^ We found that the t-nanoEM analysis should also be powerful for these regions. For example, the HSPA1A and HSPA1B genes, both belonging to the Hsp70 gene family, are highly homologous to each other.^20^ While the short EM-seq reads are difficult to discriminate these regions (Figure 3A), we found that both genes were sufficiently covered by the t-nanoEM reads. The results indicated that the promoter of the HSPA1A gene in MB231 showed increased methylation compared with that in BT474. Similarly, lower RNA expression was detected in MB231 (6 and 28 reads per kilobase of exon per million mapped reads [rpkm] in MB231 and BT474, respectively). However, HSPA1B gene promoter was completely unmethylated in both MB231 and BT474. Triple-negative breast cancers generally show lower expression of HSPA1A compared with the luminal type.^21^ A similar advantage was observed for the MUC1 gene, which encodes mucin and contains a GC-rich tandem repeat region (Figure 3B).^22^ The MUC1 gene is regulated by DNA methylation in some breast cancer cell lines based on the results of Sanger sequencing.^23^ In fact, we found that the region was methylated in BT474, but not in MB231, and MUC1 expression in MB231 was higher compared with that in BT474. This method complements the shortcomings of previous short-read-based methylation analysis methods, in which it was difficult to establish methylation profiles, even for the representative genes (Figures 3A and 3B).

### Detection of allele-biased methylation patterns from converted long reads

Using t-nanoEM, we analyzed the haplotype-biased methylation patterns for the target regions (the methylome panel regions). We separated the t-nanoEM reads by heterozygous single-nucleotide polymorphisms (hetero SNPs) considering the conversion pattern of EM-seq (Figure S2H). In theory, because the converted long reads of nanoEM and t-nanoEM often cover multiple hetero SNPs, it should be possible to reconstruct the patterns of multiple hetero SNPs (SNP phasing) by the converted long reads. Because the reads also represent the methylation patterns, these building blocks should serve as the bases for methylation profiling (methylation phasing). However, no bioinformatics tool was available for a phasing analysis that could process the converted reads. Therefore, we developed a pipeline based on a representative software for the SNP phasing known as WhatsHap (Figure S2I). Briefly, for each hetero SNP position, which was determined by the short-read whole-genome sequencing (WGS) in our previous study,^5^^,^^24^ reference or alternate bases were searched in the converted reads considering the conversion patterns of EM-seq (Figure S2H). Based on this information, pseudo reads containing reference or alternate bases in the SNP positions, which reverted to their original bases if they had been base-converted, were generated. The pseudo reads harboring the hetero SNPs were used as input for WhatsHap^25^ to construct haplotype blocks.

To evaluate the constructed phasing patterns of the SNPs, we compared them with one that was similarly established by WhatsHap but using the WGS data for native DNA sequencing on PromethION. In addition, we also performed phasing analysis using whole-genome nanoEM. The “switch error rates,” the rates of SNPs sorted to different alleles, were similar for 50, 10, and 1 ng of input for nanoEM and 10 ng of input for t-nanoEM using MB231 (0.88%, 0.89%, 1.1%, and 1.1%, respectively). They were also at a reasonable level compared with previous studies using PacBio long-read data^25^ considering that errors would be contained in the phasing patterns constructed by nanopore WGS used as comparison data (Table S1B). Although the t-nanoEM of BT474 exhibited a somewhat higher switch error (1.9%) compared with those of MB231, it would result from high aneuploidy of BT474.^26^ While the average lengths of the phased blocks in nanoEM (10–22 kb) and t-nanoEM (7.6–9.4 kb) were shorter compared with those in nanopore WGS (400–550 kb), this was simply because of the shorter read length in nanoEM and t-nanoEM^27^ as well as the focused distribution of the reads in t-nanoEM.

To detect the allele-biased DMRs, the software metilene^28^ was used (Figure 3C; Tables S1C, S2A, and S2B). We identified a total of 466 and 297 possibly allele-biased methylated regions from the t-nanoEM data of MB231 and BT474, respectively. These regions included well-known imprinting regions, such as PARD6G-AS1 and MEST (Figures 3D and 3E).^29^^,^^30^ Interestingly, allelic methylation in the imprinting region of the MEST gene was lost by demethylation only in BT474, whereas MEST expression in BT474 was higher compared with that in MB231. The imprinting of MEST is frequently lost in invasive breast cancer.^31^ This phenomenon, known as the loss of imprinting, is frequently observed in various cancers.^32^

### Application of t-nanoEM to clinical samples

To demonstrate its applicability for clinical samples, t-nanoEM was performed on fresh frozen clinical samples, including one case of breast cancer (BRC26) and one case of lung adenocarcinoma (LUAD14). Using frozen sections for each cancer tissue after methanol fixation and hematoxylin and eosin (H&E) staining, 4–5 areas, defined by a pathological view of the H&E staining or spatial transcriptome data obtained with Visium, were dissected. The specimens were subjected to t-nanoEM with the custom human methylome panel and the pan-cancer panel for breast and lung cancer, respectively.

### Application to breast cancer specimens

**Figure 4:**
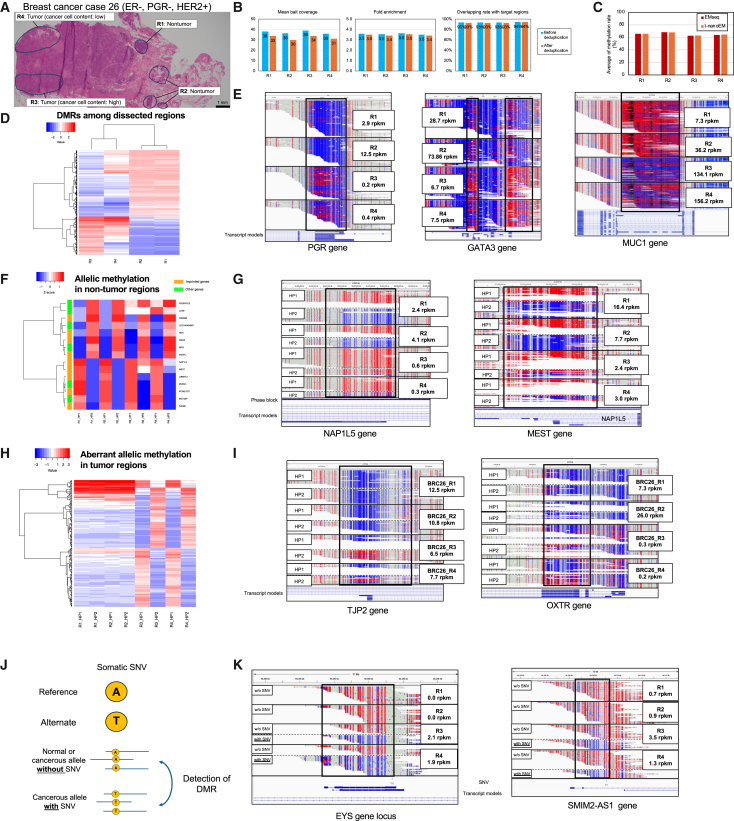
T-nanoEM analysis of the dissected breast cancer tissues (A) H&E staining image of a section of a breast cancer specimen surgically removed from a patient with breast cancer without drug treatment. The four regions enclosed by the lines were dissected. (B) The mean bait coverage, fold enrichment, and overlapping rate before and after deduplication of t-nanoEM prepared from each region with the human methylome panel (Data S1). (C) Plot showing the average of the methylation rates in CpGs covered by five reads or more common to all datasets in BRC26 as measured by short-read EM-seq and t-nanoEM. (D) Heatmap for the average CpG methylation rates in DMRs with 50% or more difference in any combination of the two regions (for details, see STAR Methods). (E) Typical views of the DMRs between nontumor and tumor regions around the PGR, GATA3, and MUC1 genes. The distribution of the t-nanoEM reads and CpG methylation status are visualized in the top panel. RefSeq transcript models are shown in the bottom panel. Sample names and expression levels of each gene measured by RNA-seq are shown in the squares. (F) Heatmap of the average CpG methylation rates in allelic methylated regions in nontumor tissues (for details see STAR Methods). The colored boxes indicate the imprinted genes registered in the Geneimprint database (https://www.geneimprint.com/). (G) Representative imprinting regions around the NAP1L5 and MEST genes were detected by filtering in (F). For each sample, the reads assigned to each haplotype and the CpG methylation status were visualized. (H) Heatmap for the average CpG methylation rates in the allelic methylated regions in the tumor tissues (for details, see STAR Methods). (I) Haplotype-specific methylation detected only in the tumor regions around the TJP2 and OXTR genes. For each sample, the reads assigned to each haplotype and the CpG methylation status were visualized. (J) Schematic view of t-nanoEM phasing by SNVs and the detection of DMRs between alleles with and without SNVs. Among the t-nanoEM reads covering the positions of the SNVs, those harboring the reference base and SNV were distinguished by considering the base conversion and were separately collected. The DMRs between the SNV and non-SNV reads were called. (K) Examples of DMRs on alleles with SNVs around the EYS and SMIM2-AS1 genes. For each sample, the reads assigned with and without SNV and the CpG methylation statuses are visualized in the top panel. Regarding R1 and R2, SNV reads were not detected in these loci. SNVs are shown in the bottom panel. See also Tables S1 and S2 and Data S1.

First, we applied t-nanoEM to breast cancer tissues because we had already optimized it in two breast cancer cell lines. For the breast cancer specimen (estrogen receptor [ER]-negative, PGR-negative, and HER2-positive: see Figure S4A for more details), four regions were defined by the pathological diagnosis based on the H&E images, including two nontumor regions (R1 and R2) and two tumor regions (R3 and R4) (Figure 4A). The R3 region exhibited a higher content of cancer cells, whereas the R4 region had a lower content of cancer cells. The multi-omics data for these regions were newly acquired for this study, ensuring a consistent dataset. These regions were dissected, and DNA and RNA were extracted and subjected to a multi-omics analysis, which involved short-read WGS, RNA sequencing (RNA-seq), and EM-seq on Illumina sequencer (Tables S1D–S1F). In addition, t-nanoEM was applied to the DNA (Table S1A). Using 14–26 ng of the DNA, t-nanoEM libraries were constructed using the custom methylome panel and sequenced using a single flow cell/sample on PromethION. 7.6–8.8 M reads at an average length of 4.6–4.9 kb were obtained. The mean bait coverage, fold enrichment, and overlapping rate with the target regions were 36–38, 3.5–3.6, and 93%–94% before deduplication and 30–35, 3.4–3.5, and 93%–94% after deduplication, respectively (Figure 4B), which were expected based on the analysis of the cell lines (Figure S3B). The average methylation rates were similar between t-nanoEM and short-read EM-seq (Figure 4C). Pearson’s correlation coefficients for t-nanoEM and EM-seq (R = 0.87–0.91) were slightly lower compared with those from the cell line data (Figures S4B and S3D). This may have occurred because the methylation rates tend to fluctuate because of cell heterogeneity in the tissue samples and the lower sequencing depth of the short-read EM-seq, particularly in R3 (×11) and R4 (×13), compared with the cell line data (×33 and 50) (Table S1F).

Consistent with the pathology results, CpG methylation rates within nontumor areas (R1 and R2; R = 0.90) or tumor areas (R3 and R4; R = 0.90) measured by the t-nanoEM data were highly correlated, whereas those between nontumor and tumor areas were lower (R = 0.75–0.81) (Figure S4C). The same trends were observed in the short-read EM-seq and RNA-seq data (Figures S4D and S4E). To further analyze the area-characteristic methylation profiles, we extracted 8,928 DMRs that differed by 50% or more in any combination of the four regions, including that of R1 and R2, and in which the CpG methylation level could be measured in all regions (Figure 4D; Table S2C). Based on this approach, both region-specific tumor methylation and demethylation regions were detected. R3, which had a high cancer cell content, exhibited the most distinct methylation patterns. For example, the PGR and GATA3 genes concordantly showed a higher methylation pattern, resulting in a lower RNA expression in the tumor regions (Figure 4E). The downregulation of PGR at the protein level, which is an important diagnostic marker, was confirmed by immunohistochemistry (Figure S4A). GATA3 is also a key transcription factor,^33^ whose functional loss is associated with tumor progression in breast cancer.^34^ Both the PGR and GATA3 genes are regulated by DNA methylation.^5^^,^^35^^,^^36^ In addition, t-nanoEM detected tumor region-specific demethylation in the MUC1 gene, which was not detected by short-read EM-seq (Figures 4E and 3B). MUC1 expression was also upregulated in tumor areas, which may play a role in the malignant phenotype of cancer in this region.^23^^,^^37^

Methylome-wide haplotype phasing and detection of DMR between haplotypes were also conducted on the breast cancer data. To expedite the comparison of the same haplotype across the regions, phasing of hetero SNPs was only performed for R2, and the phased blocks in R2 were used for allele separation of the t-nanoEM reads for all samples (Table S1G). As a result, 600 (R1), 554 (R2), 1,684 (R3), and 1,761 (R4) genomic regions were determined to be haplotype-biased DMRs, and, by merging them, 3,560 DMRs were detected (Tables S1C and S2D). Fifteen DMRs exhibited a difference between alleles by >50% in the normal regions (R1 and R2) and were located within ±10 kb from the TSS (transcription start site) of the RefSeq transcript (Figure 4F). Among the DMRs, 9 were located in the vicinity of known imprinting genes. By comparing the DMRs in nontumor and tumor regions, loss of imprinting around the MEST and NAP1L5^38^^,^^39^ genes was observed. The expression of NAP1L5 and MEST in the tumor regions was decreased, for which the methylation of the originally unmethylated allele was responsible (Figure 4G). Interestingly, inspection of the data indicated that the haplotype-specific methylation occurred more frequently in the tumor regions (Table S1C). We identified the 180 DMRs flanking the genes for which the difference between haplotypes in nontumor regions R1 and R2 was less than 10% and was 40% or more in tumor regions R3 and R4, respectively (Figure 4H). For example, the TJP2 and OXTR genes showed haplotype-biased methylation upregulation, resulting in the downregulation of their mRNAs in the tumor regions (Figure 4I). TJP2, also known as ZO-2, is a tight junction protein and a known tumor suppressor gene.^40^^,^^41^ OXTR is an oxytocin receptor, which exhibits lower expression in ER-negative tumors compared with ER-positive tumors.^42^

Even after dissection, the samples isolated from the tumor regions may still contain normal cell DNA, including that of epithelial cells and other stromal cells. To enrich the methylation information specifically for the cancer cell DNA, we attempted to isolate cancerous reads with somatic nucleotide mutations (SNVs). First, we called SNVs from the short-read WGS data. Because the R2 region was the most distant from the tumor area and considered to represent normal tissue, R2 data were used as a control for SNV calling for each sample (Figure 4A; Table S1H). By merging the SNVs called from each sample, we obtained a total of 19,622 SNVs. Similar to the haplotype/methylation phasing, the converted reads with and without SNVs were separately counted (Figure 4J). As a result, 14–61 of the DMRs were called for each sample with and without SNVs, and, by merging them, 108 DMRs were detected (Tables S1I and S2E). These regions included the FEZF2, MYOD1, and NEFL genes (Figure S4F). These genes are tumor suppressors, and their expression is regulated by DNA methylation.^43^^,^^44^^,^^45^^,^^46^^,^^47^^,^^48^ With respect to FEZF2, reads with SNV showed a significantly higher methylation status compared with those without SNV. Consistently, its expression level was downregulated in the tumor regions compared with the nontumor regions. The MYOD1 and NEFL genes were not expressed in either the nontumor or tumor regions; however, a high methylation level in the reads with SNV was detected only in the tumor areas. A similar pattern was observed for several previously uncharacterized genes. For the EYS and SMIM2-AS1 genes, low methylation was detected in the reads harboring SNVs (Figure 4K). Lower expression of these genes was observed in the tumor regions. Thus, the detected SNVs themselves may impose a causative effect on the disordered methylation (see Figure S4F for further discussion). Taken together, the results indicate that t-nanoEM provides a robust method to extract precise methylation information for cancers and identify candidate regulatory mutations.

### Application to lung cancer samples

**Figure 5:**
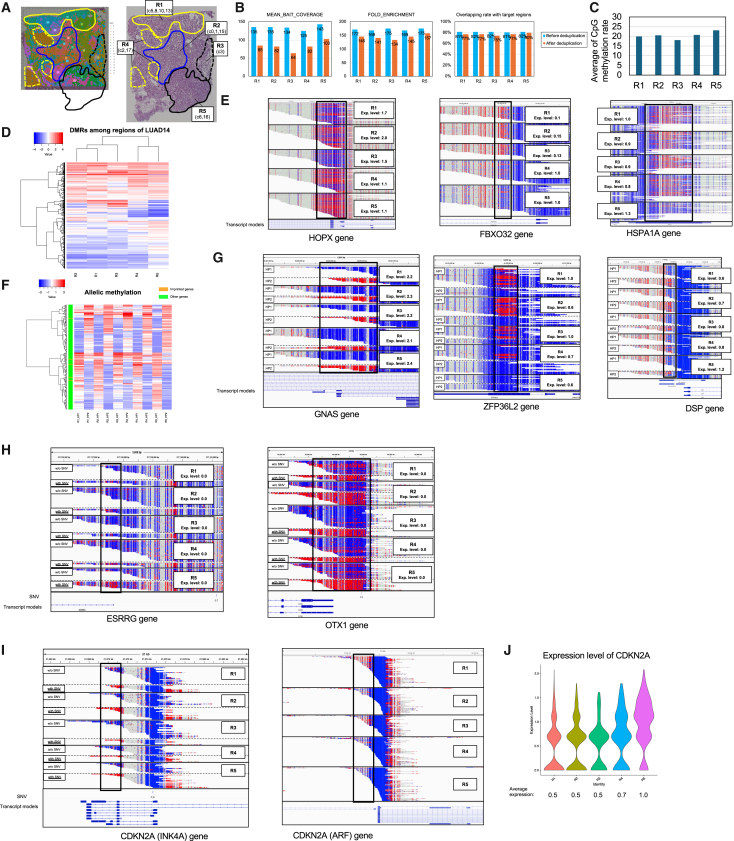
T-nanoEM analysis of the dissected lung cancer tissues (A) H&E staining images of adjacent sections of the lung cancer specimen LUAD no. 14 (LUAD14). In the left panel, the section used for the spatial transcriptome analysis with Visium v.1^49^ is shown. Each cluster constructed from the Visium data from our previous study was visualized on the H&E staining image of the section. In the right panel, a section adjacent to the Visium section is shown. Serial sections of this sample were used for microdissection. Areas corresponding to dissected regions were circled by lines. The name of each region and the cluster IDs belonging to each region are shown in the squares. (B) Mean bait coverage, fold enrichment, and overlapping rate before and after deduplication of t-nanoEM prepared from each region with a pan-cancer panel. (C) The plot of the average of the methylation rates in CpGs covered by five reads or more common to all datasets in LUAD14 measured by t-nanoEM. (D) Heatmap for the average CpG methylation rates in DMRs with 30% or more difference in any combination of the two regions (for details, see STAR Methods). (E) Typical views of the DMRs between differentiated and poorly differentiated regions around the HOPX, FBXO32, and HSPA1A genes. The distribution of the t-nanoEM reads and the CpG methylation status are visualized. The average expression levels of each gene measured by Visium are shown in squares. (F) Heatmap for the average CpG methylation rates in haplotype-specific methylated regions (for details, see STAR Methods). Imprinted genes registered in the Geneimprint database are indicated by colored boxes. (G) Representative haplotype-specific methylated regions around the GNAS, ZFP36L2, and DSP genes. The GNAS and ZFP36L2 genes are known imprinted genes. (H) Examples of DMRs on alleles with SNVs around the ESRRG and OTX1 genes. (I) DMRs around CDKN2A. In the left panel, the methylated region, where the allele with SNV is specifically methylated, around the TSS of INK4A, which is a splicing isoform of the CDKN2A gene, is shown. In the right panel, the DMR between the differentiated and poorly differentiated regions around the TSS of ARF, which is another splicing isoform of CDKN2A, is shown. No SNV was located in this locus. (J) The expression levels of the CDKN2A gene are shown. Clusters in each region were merged. The violin plots illustrate the frequency distribution of the expression levels for each Visium spot. The average expression levels for each sample are shown at the bottom. See also Tables S1 and S2 and Data S1.

To demonstrate the applicability of t-nanoEM to various tumors and the utility of multiplexing in clinical samples, we also applied this technique to a lung cancer specimen. For the lung cancer specimen, spatial transcriptomics analysis using Visium was performed, and 5 regions were defined based on pathological analysis and clusters detected by the Visium data in our previous study (Figure 5A).^49^ The clusters in R1, R2, and R3 were characterized by higher expression levels of lineage-specific markers of alveolar epithelial cells, including HOPX, SFTPA1, SFTPB, and SFTPC (Figures S5A and S5B).^50^ The clusters in R4 and R5 were characterized by higher expression levels of genes associated with hypoxia and cancer malignancy, such as VEGFA, SLC2A1, TNC, and HMGA1.^51^^,^^52^^,^^53^^,^^54^ The R4 clusters showed relatively higher and lower expression of lineage markers of alveolar epithelial cells and cancer malignancy, respectively, compared with R5. Therefore, we determined that R1, R2, and R3 were in a differentiated state, R5 was the most poorly differentiated, and R4 was in an intermediate state.

For the lung cancer samples, we used the pan-cancer panel targeting loci with cancer-specific methylation status (Figure S4G). Using 8–49 ng of gDNA extracted from each region, t-nanoEM libraries with different index sequences were prepared. After pooling the five libraries, target enrichment with the pan-cancer panel and sequencing with a single PromethION were performed. After demultiplexing with the index sequences, a similar number of reads (∼536–607 k reads) with ∼5 kb of N50 length were obtained for each region (Figure S5C; Table S1A). The mean bait coverage, fold enrichment, and overlapping rate with the target regions were 125–143, 169–175, and 81%–82% before deduplication and 64–103, 134–146, and 75%–80% after deduplication, respectively (Figure 5B). The results indicate that t-nanoEM can also be conducted with lung cancer specimens.

R5 and R4 were more poorly differentiated compared with R1–R3 and in an intermediate status based on the spatial transcriptomic data, respectively. The average of the CpG methylation rate in R5 was higher compared with that in the other regions (Figure 5C). R4 exhibited a high correlation (R = 0.85–0.88) with all regions, whereas R5 showed the highest correlation (R = 0.88) with R4 (Figure S5D). To further examine the area-characteristic methylation profiles, we extracted 962 DMRs that differed by 30% or more in any combination of the five regions and in which the CpG methylation level could be measured in all regions (Table S2F). Also, from the hierarchical clustering of the DMRs, R4 and R5 showed a different methylation pattern compared with the other regions (Figure 5D). In R4 and R5, a lineage marker of the lung epithelium HOPX exhibited higher methylation and lower expression compared with the others^55^ (Figures 5E and S5E). FBXO32 and HSPA1A showed lower methylation and higher expression in R4 and R5. It is known that FBXO32 promotes the epithelial-mesenchymal transition in cancer including lung adenocarcinoma.^56^^,^^57^ Furthermore, HSPA1A, whose methylation status is difficult to detect by short reads, may promote the malignant progression of lung adenocarcinoma.^58^

Next, we detected haplotype-specific methylation. Using the lung cancer specimen, short-read WGS and nanopore WGS of bulk tumor tissues were also performed (Tables S1D and S1J). Phasing of hetero SNPs called from the short-read WGS was done using the nanopore WGS as described in our previous study^27^^,^^59^ (Table S1G). T-nanoEM reads were phased to each haplotype using the phased patterns constructed from nanopore WGS. As a result, 193 (R1), 199 (R2), 105 (R3), 245 (R4), and 347 (R5) genomic regions were identified as haplotype-biased DMRs (Table S1C). Among the total of these 644 DMRs after merging (Table S2G), 522 were located within 10 kb of the TSS for the following analyses. Moreover, 180 DMRs showed a difference between alleles by 50% or more in at least one region (Figure 5F). Of these DMRs, 5 were located in the vicinity of the known imprinting genes, ZFP36L2, KLF14, PEG10, DLX5, and GNAS (Figures 5F and 5G). By comparing the DMRs in the differentiated and poorly differentiated tumor regions, loss of imprinting around the ZFP36L2 gene was observed, although its expression level was not much different among the regions (Figures 5G and S5E). The DSP gene showed haplotype-specific methylation in the differentiated regions, and its haplotype-specific methylation decreased in the poorly differentiated regions. Consistently, the expression DSP was upregulated in the poorly differentiated regions. Although DSP is a known tumor suppressor, its expression level is regulated by DNA methylation.^60^

In LUAD14, SNVs were called from short-read WGS and 8–39 of the DMRs were called for each sample with and without SNVs, and, by merging them, 75 DMRs between t-nanoEM reads with and without SNVs were detected (Tables S1I and S2H). For example, in ESRRG, OXT1, and CDKN2A, high methylation was observed in the reads with SNVs (Figures 5H and 5I). Downregulation of OXT1 induces proliferation, migration, and invasion in non-small cell lung cancer lines.^61^ ESRRG is a known tumor suppressor in various cancer types.^62^^,^^63^ OXT1 and ESRRG genes were rarely expressed in all of the regions (Figure S5E). CDKN2A is a well-known tumor suppressor gene and has several splicing variants with different first exons. The longer and shorter isoforms of CDKN2A encode ARF and INK4A, respectively.^64^ On the allele with SNV, the locus around the TSS of INK4A was specifically methylated (Figure 5I). Although the CDKN2A expression was increased in R5 (Figure 5J), the methylation status around the TSS of INK4A was nearly unchanged among the dissected regions. Considering the lower methylation around the TSS of ARF in R5 compared with that in the differentiated regions, the increased expression may reflect that of ARF. The expression level of ARF is positively regulated by the mitogen-activated protein kinase (MAPK) signaling pathway, which is downstream of KRAS.^65^ Interestingly, KRAS expression was upregulated in R5 (Figure S5E). Although the details remain unclear, it is possible that KRAS indirectly regulates ARF expression via demethylation. In either of the cases, these results demonstrate the power of the barcode-assisted multi-regional DNA methylation analysis by this method.

## Discussion

In this study, we developed a method for targeted long-read methylation analysis, t-nanoEM, which can be implemented with a minimum of 8 ng of gDNA. T-nanoEM demonstrated high enrichment rates (up to ×170) and coverage (up to ×570) for target regions. Moreover, it enabled the detection of methylation status in various regions, such as repetitive sequences, which are challenging to analyze with short-read sequencing. In both cancer types, we observed significant methylation differences in regions around cancer-related genes, such as MUC1 and HSPA1A,^37^^,^^58^ in which it is difficult to detect methylation status with short-read sequencing (Figures 4E and 5E). Conventional short-read methylation analysis may have overlooked these changes. Therefore, designing capture panels to these difficult-to-analyze regions, t-nanoEM can potentially reveal new insights. Regarding the clinical tissues used in this study, we dissected 10-μm-thick tissue sections. Because the samples were significantly smaller than typical tissue samples obtained by needle biopsies, t-nanoEM should be applicable to biopsy specimens.

To reduce reagent costs for hybridization capture and sequencing and increase experimental throughput, multiplexing of t-nanoEM before hybridization capture was successfully conducted (Figures 5 and S3K). For the lung adenocarcinoma, we conducted multiplexing of five libraries and hybridization capture with the pan-cancer panel covering 1.5 Mb of genomic regions and obtained about ×80 coverage (Figure 5). The results suggest that multiplexing of tens of libraries would be feasible for lower-coverage sequencing or smaller capture panels. While t-nanoEM itself is relatively labor intensive, its capability of multiplexing allows efficient processing of numerous samples simultaneously. Additionally, the original nanoEM library can be used to re-capture by another panel, further reducing input DNA. Although the yield of t-nanoEM using the premade pan-cancer panel (14 Gb) is lower compared with typical flow cell yields (Table S1A), it could be improved using a custom pan-cancer panel optimized for long-read capture (Figures S3L–S3R). The manufacturer’s information indicated that a flow cell for MinION and GridION has one-sixth the yield of a flow cell for PromethION.^66^ Thus, we expect to achieve a mean bait coverage of 100x when using the custom pan-cancer panel on a flow cell for MinION and GridION. Therefore, t-nanoEM should also apply to the flow cells for MinION and GridION.

Furthermore, we constructed a workflow for haplotype phasing using the converted long reads of nanoEM or t-nanoEM, allowing to detect haplotype-specific methylation patterns. Due to the high-depth sequencing of t-nanoEM, it detected more haplotype-specific methylated regions than whole-genome nanoEM (Table S1C). Previous studies have used long-read sequence of native DNA for haplotype-specific methylation analysis.^4^^,^^27^^,^^67^ However, the long-read data were obtained from micrograms of input DNA. By leveraging its ability to analyze lower DNA inputs, we applied t-nanoEM to local regions on breast and lung cancer tissue sections, guided by pathological views or spatial transcriptome data (Figures 4 and 5). T-nanoEM can detect not only aberrations in haplotype-specific methylation, including imprinting regions and cancer-related genes, but also the methylation status of cancerous alleles with somatic mutations. The somatic mutations in regulatory regions occasionally influence expression of their target genes.^68^ As suggested in Figure S4F, t-nanoEM can potentially detect regulatory mutations. Tumor tissues are heterogeneous in tumor cells themselves and are also composed of various cell types. In this study, we employed microdissected local regions of tumor tissues to detect the methylation status of alleles harboring somatic SNVs. Given the high-depth sequencing achievable with t-nanoEM, extracting reads derived from DNA with SNVs should be possible even from more heterogeneous samples, such as biopsy samples, especially when using smaller target panels. This capability could be valuable for patient diagnosis and stratification. While our current approach used SNVs for read separation, cancerous reads can also be effectively separated by structural variants (SVs). As shown in Figure S3S, the methylation status around SVs can be clearly detected. This offers an effective way to evaluate the influence of SVs on DNA methylation and explore their intricate relationships.

Base conversion-based methods for DNA methylation detection, such as ten-eleven translocation (TET)-assisted pyridine borane sequencing (TAPS)^69^ and direct methylation sequencing (DM-seq),^70^ other than EM-seq and bisulfite sequencing (BS-seq), are available. TAPS and DM-seq both convert methylated C (mC) but not unmethylated C (umC) to T. In mammals, most Cs in non-CpG contexts are unmethylated, meaning that the majority of Cs retains the original sequence after the conversion from mC to T. Therefore, it is possible that the conversion from mC to T in TAPS and DM-seq makes target capture more efficient and reduces the number of probes for capture, compared to the conversion from umC to T. Although it is unclear whether DM-seq can apply to long-read applications, TAPS is applied to whole-genome methylation sequencing using long read, referred to as wglrTAPS.^71^ However, its N50 read length (∼3.5 kb) is shorter than that of nanoEM (>∼5 kb), and its correlation with BS-seq (R = 0.66–0.69) is lower than nanoEM (R = 0.84–0.90 in our previous study^5^). The read length and accuracy of the methylation detection of the input library affect the scores after target capture. Therefore, nanoEM and t-nanoEM would be superior to wglrTAPS to obtain longer read length and more accurate estimation of methylation.

The hybridization probes used for t-nanoEM are designed to detect both fully converted DNA and nonconverted DNA. For TAPS, methylated C is converted to T.^69^ For five-letter seq available from Biomodal,^72^ which can simultaneously detect methylation and genetic variants, the bisulfite method is used to convert the original fragment containing mC linked by a hairpin adapter and the copied fragment not containing mC. Therefore, we believe that the target capture method optimized for t-nanoEM is also applicable to TAPS and five-letter seq, although alterations to the blocking oligos, which inhibit hybridization between libraries, may be required.

### Limitations of the study

T-nanoEM has several limitations. Firstly, due to PCR amplification constraints, it produces shorter read lengths (∼5 kb) compared to methods employing native DNA sequencing, such as Cas9-based approaches and adaptive sampling.^8^^,^^9^ For applications requiring longer read lengths, such as methylation analysis of repetitive sequences exceeding 10 kb, these alternative methods might be more suitable, especially when several micrograms or hundreds of nanograms of DNA are available. Secondly, base-conversion approaches inherently limit the ability to distinguish certain SNP and SNV patterns (Figure S2H). This can somewhat diminish the efficiency of read phasing by variants. Additionally, t-nanoEM requires a more complex workflow, involving nanoEM library preparation, hybridization capture, and nanopore sequencing library preparation. This translates to increased labor and time requirements compared to other methods. Moreover, for tissue section analysis, t-nanoEM requires fresh frozen samples. Although we also examined its application to formalin-fixed paraffin-embedded (FFPE) samples (Figure S1J), the library size was limited to only ∼1 kb. In the present study, we only performed t-nanoEM on a limited number of clinical samples. Although the primary aim was the development of t-nanoEM and the evaluation of its applicability to clinical samples, we plan to apply t-nanoEM to larger cohorts in the future.

While t-nanoEM demands the smallest input amount among current targeted long-read methylation methods, it still requires nanograms of DNA due to the limitations of EM-seq.^5^^,^^73^ Recently, advancement in an enzymatic conversion method have enabled short-read DNA methylation analysis from as little as 10 pg of input DNA, the amount present in a single human cell.^74^ Adapting this method to t-nanoEM could potentially enable long-read methylation analysis at the single-cell level and application to spatial analysis in single-cell resolution.

## Resource availability

### Lead contact

Requests for further information and resources should be directed to the lead contact, Masahide Seki (mseki@edu.k.u-tokyo.ac.jp).

### Materials availability

Oligonucleotide sequences of the PCR primers are available in Table S3.

### Data and code availability

The cell line data were deposited in DDBJ Sequence Read Archive (https://www.ddbj.nig.ac.jp/dra/index-e.html) under the accession number DRA: PRJDB18866. The datasets of clinical samples were deposited in Japanese Genotype-phenotype Archive (https://www.ddbj.nig.ac.jp/jga/index-e.html) under the accession numbers JGA: JGAS000758 and JGA: JGAS000757 for the breast cancer and the lung adenocarcinoma, respectively. The scripts for demultiplexing and phasing analysis using the converted long reads have been archived on Zenodo via their respective DOIs (Zenodo: https://doi.org/10.5281/zenodo.14219470 and Zenodo: https://doi.org/10.5281/zenodo.17111695). The scripts are also available at the GitHub repositories (https://github.com/masahide-seki/longread_demux and https://github.com/masahide-seki/t-nanoEM). Any additional information required to reanalyze the data reported in this work paper is available from the lead contact upon request.

## Acknowledgments

We thank Kazumi Abe, Mari Tsubaki, Kiyomi Imamura, and Kunio Harada for assistance with the experiments and Erina Ishikawa and Risa Fujinaga for assistance with the data processing and analyses. We appreciate the support of Twist Bioscience in designing the capture probes and their technical advice. This study was supported by 10.13039/501100001691JSPS KAKENHI grant numbers JP21K15074 (to M.S.) and JP23H02467 (to M.S.); MEXT KAKENHI grant numbers JP22H04925 (PAGS) (to Y. Suzuki), JP20H05906 (to M.S.), and JP20H05905 (to M.S.); and the 10.13039/100009619Japan Agency for Medical Research and Development (AMED) GRIFIN grant number JP25tm0424235 (to M.S.), ASPIRE grant number JP23ama221522 (to Y. Suzuki) and P-PROMOTE grant number JP23ama221522 (to A.S.). The super-computing resource was provided by Human Genome Center, the Institute of Medical Science, The University of Tokyo. The authors thank Enago (www.enago.jp) for the English language review.

## Author contributions

Conceptualization, Y. Suzuki and M.S.; methodology, S.Z. and M.S.; software, Y. Sakamoto, Y.K., and M.S.; formal analysis, K. Kunigo, K. Kajiya, A.S., and M.S.; investigation, K. Kunigo, S.N., S.Z., A.K., A.S., and M.S.; resources, S.N., K.N., M.T., G.I., A.M., K.T., M.C., J.K., and A.S.; data curation, M.S.; writing – original draft, K. Kunigo, Y. Suzuki, and M.S.; visualization, K. Kunigo and M.S.; supervision, Y. Suzuki and M.S.; project administration, Y. Suzuki and M.S.; funding acquisition, A.S., Y. Suzuki, and M.S.

## Declaration of interests

The authors declare no competing interests.

## Declaration of generative AI and AI-assisted technologies in the writing process

During the preparation of this work, the authors used Gemini (https://gemini.google.com) for manuscript proofreading. After using this tool, the authors reviewed and edited the content as needed and take full responsibility for the content of the publication.

## STAR★Methods

### Key resources table

**Table:** 

REAGENT or RESOURCE	SOURCE	IDENTIFIER
**Critical commercial assays**		

NEBNext EM-seq Kit for Twist Targeted Methylation Sequencing	Twist Bioscience	101976
KOD ONE (KOD Xtreme Hot Start DNA Polymerase)	TOYOBO	KMM-101
ProNex Size-Selective DNA Purification System	Promega	NG2001
Twist Methylation Enhancer	Twist Bioscience	103557
Twist Universal Blocker	Twist Bioscience	100578
Twist Standard Hyb and Wash Kit v.2	Twist Bioscience	104446
Twist Binding and Purification Beads	Twist Bioscience	100984
The Twist Alliance Pan-cancer Methylation Panel	Twist Bioscience	104695
Customized human methylome panel	Twist Bioscience	MTE-99106387
Customized pan-cancer panel	Twist Bioscience	MTE-93035895
M-270 Streptavidin Dynabeads	Thermo Fisher Scientific, Inc.	65306
KOD FX *Neo*	TOYOBO	KFX-201
Ligation Sequencing Kit V14	Oxford Nanopore Technologies, Inc.	SQK-LSK114
PromethION Flow Cell	Oxford Nanopore Technologies, Inc.	FLO-PRO114M

**Deposited data**		

Raw data	This paper	DRA: PRJDB18866 JGA: JGAS000758 JGA: JGAS000757
NanoEM v1 data	Sakamoto et al.^5^	DRA: PRJDB10864
Spatial transcriptome data	Takano et al.^49^	https://kero.hgc.jp/Ad-SpatialAnalysis_2024.html

**Experimental models: Cell lines**		

MDA-MB-231	ATCC	HTB-26
BT-474	ATCC	HTB-20

**Oligonucleotides**		

See Table S3 for the PCR primers	This paper	N/A

**Software and algorithms**		

nanoEM pipeline	Sakamoto et al.^5^	https://github.com/yos-sk/nanoEM
t-nanoEM pipeline	This paper	Zenodo: https://doi.org/10.5281/zenodo.17111695
Demultiplexing pipeline	This paper	Zenodo: https://doi.org/10.5281/zenodo.14219470
Porechop v0.2.4	Wick et al.^75^	https://github.com/rrwick/Porechop
minimap2 v.2.17	Li.^76^	https://github.com/lh3/minimap2
Samtools v.1.7	Danecek et al.^77^	https://github.com/samtools/samtools
Picard v.3.1.0	Broad institute	https://broadinstitute.github.io/picard/
WhatsHap v.1.0	Martin et al.^25^	https://whatshap.readthedocs.io/en/latest/index.html
metilene v.0.2-8	Jühling et al.^28^	http://legacy.bioinf.uni-leipzig.de/Software/metilene/
HOMER v.5.0.1	Heinz et al.^78^	https://github.com/javrodriguez/HOMER

### Experimental model and study participant details

#### Clinical specimens

Informed consent was obtained from a breast cancer patient (80-years old, female) at St. Marianna University School of Medicine Hospital. This study was approved by the Clinical Ethics Committee of St. Marianna University School of Medicine (IRB#: 2297-i103) and the Research Ethics Committee of the University of Tokyo (IRB#: 22–234). The fresh frozen clinical breast cancer specimen used in this study was designated case 26 (BRC26) and was ER-negative, PGR-negative, and HER2-positive (fluorescent *in situ* hybridization (FISH) 3+) as determined by histopathology and FISH analysis of the HER2 locus. For the lung cancer specimen, informed consent was obtained from the patient (61-years old, male) at the National Cancer Center Hospital East in Japan. This study was also approved by the Clinical Ethics Committee of the National Cancer Center (IRB#: 2020-187) and the Research Ethics Committee of the University of Tokyo (IRB#: 20–164). The fresh frozen clinical lung cancer specimen and the FFPE block of its normal counterpart were obtained from the National Cancer Center Hospital East. LUAD No. 14 (LUAD14) was diagnosed as lung adenocarcinoma based on histopathology and was identical to the specimen with the same name described in our previous study.^49^

#### Cultivation of human breast cancer cell lines and DNA extraction

The human breast cancer cell lines, BT-474 (ATCC, HTB-20)^79^ and MDA-MB-231 (ATCC, HTB-26),^80^ were cultured and DNA was extracted as described previously.^5^

### Method details

#### Preparation of nanoEM v2 libraries for whole-genome long-read methylation

NanoEM v2 libraries were prepared based on our previous study^81^ with some modifications. Briefly, genomic DNA was fragmented using a g-tube (Covaris). End prep and adapter ligation of the fragmented DNA were performed using the NEBNext Enzymatic Methylseq Kit (EM-seq kit; New England Biolabs). Following adapter ligation, the sample was purified using 110 μL of Total Purification Beads (Twist Bioscience) with a magnetic stand for the PCR tubes, such as 10X Magnetic Separator (10X Genomics) and MagnaStand v3.2 (NIPPON Genetics). The sample was oxidized using the TET2 oxidation reagents of the EM-seq kit, purified using 90 μL of Total Purification Beads, and eluted with 6.6 μL of Elution Buffer from the EM-seq kit. Next, 13.4 μL of formamide was added to 6.6 μL of the sample and the oxidized DNA was denatured by incubation at 80°C for 10 min. APOBEC deamination of the denatured sample was done using the APOBEC reaction regents of the EM-seq kit. The deaminated DNA was purified using 100 μL of Total Purification Beads and eluted with 40 or 80 μL of Nuclease-Free Water (NFW; Thermo Fisher Scientific). The sample was amplified using KOD ONE with primers containing unique dual index sequences (Table S3) in a C1000 thermal cycler (Bio-Rad). The amplified DNA was purified and eluted with 52 μL of NFW using the DNA Clean & Concentrator-5 (Zymo Research). The purified DNA was size-selected with 0.82–0.9× volume of ProNex Size-Selective Chemistry of the ProNex Size-Selective DNA Purification System (Promega).

#### Target enrichment for long-converted DNA

The converted long DNA derived from the regulatory regions was enriched using the Twist Standard Hyb and Wash Kit v2, Twist Universal Blockers, Twist Methylation Enhancer, and Twist Binding and Purification Beads with the customized human methylome panel (Order ID: MTE-99106387), the Twist Alliance Pan-cancer Methylation Panel, or the customized pan-cancer panel (Order ID: MTE-93035895; Twist Bioscience) (Data S1). The protocol for long-converted DNA was developed by combining two protocols: the Twist Targeted Methylation Sequencing Protocol for short-read EM-seq (https://www.twistbioscience.com/sites/default/files/resources/2022-06/DOC-001222_Protocol_TargetedMethylationSequencing-REV4-singles.pdf) and the Long-Read Library Preparation and Standard Hyb v2 Enrichment (https://www.twistbioscience.com/sites/default/files/resources/2023-02/DOC-001320-Protocol_LongReadLP-StdHyb-v2Enrichment-REV2-SINGLES.pdf), released from Twist Bioscience with some modifications.

First, 2 μL of Methylation Enhancer and 500 ng of the nanoEM v2 library were added to a fresh 1.5 mL tube and the water in the mixture was evaporated at room temperature using an Eppendorf Vacufuge 5305 Plus Concentrator Complete System (Eppendorf). Then, 0.25 μL of customized human methylome panel and 7.75 μL of NFW or 4 μL of the Twist Alliance Pan-cancer Methylation Panel and 4 μL of NFW were added to a fresh PCR tube. The dried library was resuspended by adding 5 μL of Blocker Solution and 7 μL of Universal Blockers, and the resuspended library was transferred to a fresh PCR tube. The probe mixture was incubated at 95°C for 2 min in a T100 thermal cycler (Bio-Rad) on ice for 5 min and then at room temperature for 5 min. The resuspended library was incubated at 95°C for 1 min in the thermal cycler and then at room temperature for 5 min. The entire volume of the probe mixture and 30 μL of Hybridization Enhancer were added to the library. The sample was then incubated at 70°C for ∼16 h in a thermal cycler.

Dynabeads M-270 Streptavidin (100 μL) (Thermo Fisher Scientific) was added to a fresh 1.5 mL tube and washed three times with 200 μL of Binding Buffer using a magnetic stand DynaMag-2 (Thermo Fisher Scientific). After washing, the pellet of beads was suspended in 200 μL of Binding Buffer and the suspension was incubated for over 10 min at 65°C in a heat block. After the hybridization reaction, the entire sample was added to the incubated bead suspension without removing the PCR tube from the thermal cycler. After incubating for 5 min at 65°C, the sample was placed on a magnetic stand and the supernatant was removed. The bead pellet was resuspended in 200 μL of Standard Wash Buffer 1 preheated at 65°C. After incubation for 5 min at 65°C, the suspension was transferred to a fresh 1.5 mL tube. The tube was placed on the magnetic stand and the suspension was removed. The bead pellet was resuspended in 200 μL of Wash Buffer 2 preheated at 48°C. After incubation for 5 min at 48°C, the tube was placed on a magnetic stand and the suspension was removed. The wash step with Wash Buffer 2 was performed two more times (three times in total). After the third wash, the bead pellet was suspended in 12 μL of NFW. To denature the hybridized DNA, 12 μL of 0.2 N NaOH was added to the suspension and the mixture was incubated for 5 min at room temperature. Then, 24 μL of 200 mM Tris-HCl (pH 8.0) was added to the sample. To recover the captured DNA, the tube was set on the magnetic stand and the supernatant was transferred to a fresh PCR tube.

The captured DNA was amplified using KOD FX *Neo* (TOYOBO) and amplification primers, which are part of the Twist Standard Hyb and Wash Kit v2. Next, 100 μL of 2X PCR Buffer for KOD FX *Neo*, 40 μL of 2 mM dNTPs, 6 μL of amplification primers, and 4 μL of KOD FX *Neo* were added to the sample (∼50 μL). After mixing, the PCR mixture was divided into ∼100 μL aliquots in two PCR tubes. Using a C1000 thermal cycler with a 96-deep Well Reaction module (Bio-Rad), PCR amplification was carried out with the following cycles: 1 cycle of 1 min at 94°C, 12 or 13 cycles (for the custom human methylome panel and the pan-cancer panel, respectively) of 15 s at 94°C, 30 s at 63.8°C, 15 min at 68°C, and 1 cycle of 15 min at 68°C. To purify the DNA, 50 μL of DNA Purification Beads (a component of Twist Binding and Purification Beads) were added to each tube and incubated at room temperature for 10 min. The tubes were placed on a magnetic stand for PCR tubes (10X Magnetic Separator and Magna Stand v3.2) and the supernatant was removed. The bead pellets were washed twice with 80% ethanol. After removing the remaining supernatant completely, the bead pellets were dried by incubation at room temperature for 1 min. Each dried bead pellet was suspended with 25.5 μL NFW. After incubation at 37°C for 10 min, the tubes were placed on a magnetic stand and the supernatant containing the amplified DNA was transferred to a single fresh PCR tube. For the selection of long DNA fragments, 42 μL of ProNex Size-Selective Chemistry of ProNex Size-Selective DNA Purification System was added to 50 μL of the sample and the mixture was incubated for 15 min at room temperature. After washing twice with 200 μL of Wash Buffer of the ProNex Size-Selective DNA Purification System, the bead suspension was dried by incubating for 1 min at room temperature and suspended into 52 μL of NFW. For the evaluation of the distribution of the DNA fragment length and quantitation, a DNA12000 kit with a 2100 Bioanalyzer (Agilent Technologies), and a Qubit dsDNA HS Assay Kit with a Qubit 4 Fluorometer (Thermo Fisher Scientific) were used, respectively.

#### Nanopore sequencing of the nanoEM and t-nanoEM libraries

Nanopore libraries were prepared using the Ligation Sequencing Kit V14 (SQK-LSK114, Oxford Nanopore Technologies) based on the manufacturer’s instructions. Briefly, 250–300 ng of nanoEM and t-nanoEM libraries, DNA repair, and end preparation were performed using the NEBNext FFPE DNA Repair Mix and a NEBNext Ultra II End Repair/dA-tailing module (New England Biolabs). After purifying the DNA using AMPure XP Beads (Beckman Colter), the sequencing adapter was ligated to the sample using the NEBNext Quick Ligation module (New England Biolabs). The adapter-ligated sample was purified with AMPure XP Beads and Short Fragment Buffer and eluted with Elution Buffer. The nanopore libraries were quantified using the Qubit dsDNA HS Assay Kit (Thermo Fisher Scientific). Nanopore sequencing was performed using a PromethION sequencer with an R10.4.1 PromethION flow cell (FLO-PRO114M, Oxford Nanopore Technologies). The libraries (50 ng) were mixed with Sequencing Buffer and Library Beads and loaded into the flow cell. One day after the start of sequencing, reloading of 50 ng of libraries was performed after washing with the Flow Cell Wash Kit (EXP-WSH004, Oxford Nanopore Technologies).

#### Data analysis of the nanoEM and t-nanoEM

The nanoEM or t-nanoEM reads were trimmed using parts of the adapter sequences (TGACTGGAGTTCAGACGTGTGCTCTTCCGATCT and ACACTCTTTCCCTACACGACGCTCTTCCGATCT) with the adapter trimming software Porechop v0.2.4 and the default parameter.^75^ The plots of read length were generated using NanoPlot v1.42.0.^82^ For the following analyses, we used the nanoEM pipeline developed in our previous study.^5^ PCR duplicates were removed using the MarkDuplicates function of Picard v3.1.0 (https://broadinstitute.github.io/picard/). The mean bait coverage and fold-enrichment were calculated using the CollectHsMetrics function of Picard v2.26.8. Overlapping rates were calculated from the number of overlapping reads with target regions counted using the intersect function of bedtools v2.29.0.^83^ For the premade pan-cancer panel, the bed file of the target regions was downloaded from the website of Twist Bioscience (https://www.twistbioscience.com/resources/data-files/twist-alliance-pan-cancer-methylation-panel-15mb-resource-files). Scatterplots of the methylation rate of CpG covered by five reads or more were drawn using the geom_bin_2d function of ggplot2 v3.5.0.

For phasing analysis using the t-nanoEM or nanoEM reads, scripts and their explanations are available in the GitHub repository (https://github.com/masahide-seki/t-nanoEM). When performing phasing of the heterozygous single-nucleotide polymorphisms (hetero SNPs) using converted long-reads, the bases of the converted reads on the positions with the hetero SNPs called from the short-read whole-genome sequencing (WGS) were extracted using the mpileup function of samtools v1.7. Based on the extracted information, each base was restored to its original base considering the base conversion pattern of EM-seq. Pseudo reads having restored bases were generated. However, other bases in these reads were replaced with Ns. The hetero SNPs called by the short-read WGS were phased with the bam file of the pseudo reads using the phase function of WhatsHap v1.0.^25^ To remove the effect of SVs, only primary alignment was used for haplotype phasing. Therefore, complex SVs, which are typically demonstrated as combinations of primary alignment and supplementary alignment(s), rarely affected our phasing approach. Each pseudo read was phased to either haplotype with the phased hetero SNP using the haplotag function of WhatsHap. When using the hetero SNP phased by the converted reads, whose phased length was relatively short, the pseudo reads were also separated by unphased hetero SNPs. When using those phased by nanopore WGS, only phased hetero SNPs were used as the input for haplotag. The phase information of each pseudo read was added to its original converted long read.

For the separation of the converted reads by somatic SNVs called from short-read WGS, the bases of the converted reads on the positions with the SNVs were extracted using the mpileup function of samtools. Considering the base conversion pattern of EM-seq, the presence of SNVs in each read was assessed and classified as reads with and without SNV. Therefore, regardless of their allelic frequency, we separated reads with or without SNVs and called the DMRs between them. This enabled the detection of specific methylation patterns in DNA containing SNVs versus DNA without them, including DNA from both normal cells and cancer-alleles without SNVs.

For the detection of DMRs, when comparing between samples, methylation rates of CpG sites covered by five reads or more were used as input for DMR calling with metilene v0.2-8.^28^ When comparing between alleles, methylation rates of CpG sites covered by three or more reads were used. The DMRs having a q-value <0.01 were selected. The DMRs were annotated using annotatePeaks.pl of HOMER v5.0.1^78^ and RefGenes downloaded from Illumina iGenome (https://jp.support.illumina.com/sequencing/sequencing_software/igenome.html). Regarding Figures 4D and 5D, DMR in all combinations was called using metilene. The DMRs were merged using bedtools and the average methylation rates for the CpGs covered by five or more reads within the merged DMR were estimated. Among the DMRs, in which the methylation status could be detected in all samples, the DMRs with a difference in methylation rate of 50% or more and 30% or more between samples were extracted for the breast and lung cancer tissues, respectively. Regarding Figures 4F, 4H, and 5F, the DMRs between haplotypes called for each sample were merged and the average methylation rates for the CpGs covered by three reads or more within the merged DMR were estimated. Among the haplotype-specific methylated regions, in which methylation status could be detected in all samples, those located within ±10 kb from the TSS of the RefSeq transcript were extracted. The haplotype-specific methylated regions in nontumor areas, which had a difference in methylation rate of 50% or more in both R1 and 2, were extracted (Figure 4F). Figure 4H shows the haplotype-specific methylated regions in the tumor areas with a difference in methylation rates of 40% or more in both R3 and R4, and less than 10% in both R1 and R2 were further extracted. Figure 5F shows the extracted haplotype-specific methylated regions, which exhibit a difference in methylation rate of more than 50% in any region. The heatmap of the DMRs was generated using the heatmap2 function of the gplots package v3.1.3.1 of R. Hierarchical clustering of the DMRs was done using the ward. D2 method. The gene names listed on the side of the heatmap refer to the genes whose TSS is closest to the haplotype-specific DMRs.

#### Data analysis of the multiplexed t-nanoEM

For multiplexed t-nanoEM libraries, to split reads with the internal adapter, adapter trimming was performed using Porechop^75^ with adapter sequences (AATGATACGGCGACCACCGA and CAAGCAGAAGACGGCATACGA) outside of the barcode sequences corresponding to P5 and P7 of the Illumina library. For demultiplexing, the reads with at least one barcode sequence detected within 60 bp of the end of the reads were judged as reads with the barcode using a custom script (https://github.com/masahide-seki/longread_demux). When an unexpected barcode pair was detected or the barcode was not detected, the reads were removed. Then, the demultiplexed reads were analyzed using the same workflow with the single-plex t-nanoEM data.

#### Dissection of the cancer specimens

Frozen breast cancer specimens embedded in OCT compound were sectioned at a 10 μm thickness using a cryostat and placed onto glass slides. Fixation in methanol and hematoxylin and eosin staining was done following a protocol available from 10x Genomics (https://cdn.10xgenomics.com/image/upload/v1660261285/support-documents/CG000312_Demonstrated_Protocol_Methanol_Fixation_and_IF_Staining_RevD.pdf). Dissection of the slides was performed using the AVENIO Millisect System with a Milling Tip (Roche). The dissected tissues were used for extracting DNA or RNA.

#### DNA extraction from dissected tissues

Genomic DNA was extracted from each dissected tissue using a NucleoSpin Tissue XS (MACHEREY-NAGEL) based on the manufacturer’s protocol. The size distribution and amount of extracted DNA were measured using a 2200 TapeStation with a Genomic DNA Kit (Agilent Technologies) and a Qubit 4 Fluorometer with a Qubit dsDNA HS Assay Kit (Thermo Fisher Scientific), respectively.

#### RNA extraction from the dissected breast cancer tissues

Total RNA was extracted from each dissected breast cancer tissue using a RNeasy Micro Kit (Qiagen) without carrier RNA following the manufacturer’s instructions. The extracted RNA was quantified using a 2100 Bioanalyzer and an RNA 6000 Pico Kit (Agilent Technologies).

#### RNA-seq of the dissected breast cancer tissues

RNA-seq libraries were prepared from 5 ng of total RNA extracted from each dissected tissue using an SMART-seq Stranded Kit (Takara Bio) based on the manufacturer’s instructions. Briefly, RNA was fragmented by heat treatment for 4 min. After reverse transcription of the fragmented RNA, the 1^st^ PCR reaction (five cycles) was performed. The amplified cDNA was purified using AMPure XP and cDNA derived from ribosomal RNA was cleaved by treatment with scZapR and scRProbes to prevent amplification during the second PCR reaction. The second PCR reaction was performed for 14 cycles. After purification using AMPure XP Beads, the library was quantified using a 2100 Bioanalyzer and 2100 and DNA 7500 Kit (Agilent Technologies). Paired-end sequencing (150 bp) was conducted using NovaSeq6000 (Illumina).

#### Estimation of expression from RNA-seq of the dissected breast cancer tissues

Adapter trimming of RNA-seq reads was performed using trim galore v0.6.4 (https://github.com/FelixKrueger/TrimGalore) and the “--clip_R2 3” option because the three bases at the 5′ end of Read 2 are derived from the adapter in the SMART-seq Stranded Kit. The trimmed reads were aligned to the reference genome GRCh38.p12 using STAR v2.7.3a with the following options: “--outSAMstrandField intronMotif --readFilesCommand gunzip --outSAMtype BAM SortedByCoordinate.” The reads were mapped to each gene in RefGenes downloaded from Illumina iGenome. They were counted using featureCounts v1.6.4 with the following options: “-t exon -g gene_id.” RPKM values for each gene were calculated from them.

#### Short-read EM-seq of the dissected breast cancer tissues

EM-seq libraries were prepared using the EM-seq kit based on the manufacturer’s instructions and described in our previous study.^5^ Briefly, DNA was fragmented using an M220 focused-ultrasonicator (Covaris) using the following settings: duty factor 20%, peak power 50 W, cycles/burst 200, and duration 60 s. End preparation before adapter ligation was done using NEBNext Ultra II End Prep Enzyme Mix and NEBNext Ultra II End Prep Enzyme Mix. Ligation of the NEBNext EM-seq Adaptor was performed using the NEBNext Ultra II Ligation Master Mix. After purification with NEBNext Sample Purification Beads, TET oxidation and glycosylation of the adapter-ligated DNA were carried out to protect the methylated cytosines from APOBEC conversion. After the purification of the reaction product using NEBNext Sample Purification Beads, denaturation with 20% formamide and subsequent APOBEC conversion were performed. After purification using NEBNext Sample Purification Beads, the converted DNA was amplified by nine cycles of PCR using NEBNext Q5U Master Mix and EM-seq Index Primer. After purification using NEBNext Sample Purification Beads, the library was quantified using a 2100 Bioanalyzer and a High Sensitivity DNA Kit (Agilent Technologies). Paired-end sequencing (150 bp) was done using the NovaSeq6000 (Illumina).

#### Data analysis of short-read EM-seq data of the dissected breast cancer tissues

The reads of the short-read EM-seq data were trimmed using trim galore v0.6.4_dev with the following option: “--2color 20 --paired.” The trimmed reads were mapped to the human reference genome GRCh38.p12 using Bismark v0.22.1^84^ and the “-X 1000” option. After deduplication with deduplicate_bismark of Bismark, methylation information in the CpG context was extracted using bismark_methylation_extractor of Bismark with the following options: “--ignore 11 --ignore_3prime 1 --ignore_r2 5 --ignore_3prime_r2 2 --gzip --bedGraph.”

#### Short-read WGS of the dissected breast cancer tissues

WGS libraries were prepared using the EM-seq kit without procedures for base conversion. The fragmented DNA used for the short-read EM-seq was also used for the short-read DNA-seq. End preparation prior to adapter ligation was done using the NEBNext Ultra II End Prep Enzyme Mix and NEBNext Ultra II End Prep Enzyme Mix. Ligation of the NEBNext EM-seq Adaptor was performed using the NEBNext Ultra II Ligation Master Mix. After purification with NEBNext Sample Purification Beads, the adapter-ligated DNA was amplified by nine cycles of PCR using the NEBNext Q5U Master Mix and EM-seq Index Primer. After purification using NEBNext Sample Purification Beads, the library was quantified using a 2100 Bioanalyzer and a High Sensitivity DNA Kit (Agilent Technologies). Paired-end sequencing (150 bp) was carried out using a NovaSeq6000 (Illumina).

#### Short-read WGS data analysis of the breast cancer specimen

For SNP calling, the short-read WGS was trimmed using trim galore v0.6.4_dev with default parameters. The trimmed reads were aligned to the human reference genome GRCh38.p12 using BWA-MEM v0.7.17.^85^ PCR duplicates were removed using samtools v1.7. The SNPs were called using the HaplotypeCaller of GATK v4.2.0.0 as described in our previous study.^5^

For SNV calling, short-read WGS data were mapped using BWA-MEM v0.7.17 to the human reference genome GRCh38.p12 using default parameters. The mapped reads were sorted and indexed by samtools v1.7 and duplicate reads were marked by MarkDuplicates of Picard v2.23.8. Somatic mutations were detected and filtered using Mutect2 and FilterMutectCalls of GATK v 4.1.3.0, respectively.

#### DNA extraction from lung cancer and normal specimens

DNA was extracted from the fresh frozen lung cancer specimen using the MagAttract HMW DNA Kit (Qiagen) following the manufacturer’s instructions. For the normal counterpart, only the FFPE sample was available. Therefore, we extracted DNA using the QIAamp DNA FFPE Tissue Kit (Qiagen) based on the manufacturer’s instructions. Quantification of the extracted DNA was performed using a Qubit dsDNA BR Assay Kit with a Qubit 4 Fluorometer.

#### Short-read WGS of the lung cancer and normal specimens

The gDNA of both the normal and tumor specimens was fragmented using an M220 focused-ultrasonicator (Covaris) with the following settings: duty factor 20%, peak power 50, cycles/burst 200, and duration 120 s. For the tumor sample, a WGS library was prepared from 100 ng of the fragmented DNA using a TruSeq Nano DNA Library Prep Kit (Illumina) based on the manufacturer’s instructions. For the normal sample, the library was prepared from 100 ng of the fragmented DNA using a NEBNext FFPE DNA Repair v2 module, a NEBNext Ultra II DNA Library Prep Kit, and a NEBNext Multiplex Oligos for Illumina (96 Unique Dual Index Primer Pairs) (New England Biolabs) based on the manufacturer’s instructions. Paired-end sequencing (150 bp) was done using a NovaSeq6000 (Illumina).

#### Long-read WGS of the lung cancer specimen using PromethION

A library of lung cancer for nanopore sequencing was prepared using the Ligation Sequencing Kit V14 (SQK-LSK114) following the manufacturer’s instructions. Sequencing of the libraries was done using an R10.4.1 flow cell (FLO-PRO114M) and a PromethION twice. Bam files were generated by live base-calling using the SUP mode of guppy v6.5.7. The bam file merging two runs was used.

#### Data analysis of the lung WGS datasets

The reads of short-read WGS were trimmed using fastp v0.23.2^86^ and aligned to the reference genome hg38. SNP and SNV calling were performed as described in our previous study.^59^ Phasing analysis of hetero SNPs using the nanopore native DNA sequencing was performed as described in our previous study.^27^^,^^59^

#### Data analysis of Visium data of the section of lung adenocarcinoma

Visium data of LUAD No.14 (LUAD14) obtained in our previous study^49^ was used. The raw and processed data including the Seurat object file were obtained from the Japanese Genotype-phenotype Archive under the accession number JGA: JGAS000613 (https://humandbs.dbcls.jp/en/hum0394-v1) and our website (https://kero.hgc.jp/Ad-SpatialAnalysis_2024.html), respectively. The data were processed using Seurat v4.0.0^87^ as described in our previous study.^49^ The UMI counts for each spot were normalized using the SCTtransform function of Seurat and used as expression values. The average expression levels of each region were calculated by averaging the expression values of the spots belonging to the clusters in each region. Violin plots for each gene were generated using the VlnPlot function of Seurat v5.1.0.

#### Prototype of t-nanoEM using the SureSelectXT Methyl-Seq system

1.7 μg gDNA of BT474 was fragmented to ∼10 kb using a g-tube (Covaris). After TET oxidation of the fragmented DNA using the EM-seq kit, target enrichment was performed using the SureSelectXT Methyl-Seq Reagent Kit and the Human Methyl-Seq Capture library (Agilent Technologies) without using blockers for the adapters. The target-captured DNA was eluted by probe digestion with RNase H (New England Biolabs). After the eluted DNA was purified using NEBNext Sample Purification Beads (New England Biolabs), it was converted using the EM-seq kit. Adapter ligation was performed using the Accel-NGS Methyl-Seq DNA Library Kit (Swift Biosciences) with the following modifications. In this modified protocol, denaturation of DNA was performed at 94°C for 15 s and primer extension after adaptase reaction was performed using KOD -Multi & Epi- (TOYOBO) under the following conditions: 15 s at 94°C, 2 min at 62°C, 10 min at 65°C, and then hold at 4°C. PCR amplification was carried out using KOD ONE (TOYOBO) under the following condition: 22 cycles of 15 s at 94°C, 5 s at 57°C, and 15 min at 68°C. After size-selection with 0.9 volumes of ProNEX chemistry (Promega), the library was quantified using a DNA7500 kit with a 2100 Bioanalyzer (Agilent Technologies). After library preparation for nanopore sequencing with the Ligation Sequencing Kit (SQK-LSK110), it was sequenced with a PromethION flow cell (FLO-PRO002). Base-calling was performed using the SUP model of guppy v5.0.11. 1day pass reads were aligned to the human reference genome using the nanoEM pipeline^5^ with minimap2 v2.17.

#### Prototype of t-nanoEM using the Twist Fast Hybridization and Wash Kit

200 ng of the nanoEM v2 library prepared from 10 ng of MB231 gDNA were captured by the Twist Fast Hybridization and Wash Kit with the custom human methylome panel. First 0.5 μL of the custom human methylome panel, 5 μL of Blocker Solution, 8 μL of Universal Blockers, 2 μL of Methylation Enhancer, and 200 ng of nanoEM v2 library were added to a fresh 1.5 mL tube and the water in the mixture was evaporated at room temperature using the Eppendorf Vacufuge 5305 Plus Concentrator Complete System (Eppendorf). The dried nanoEM library was resuspended by adding 20 μL of Fast Hybridization Mix preheated at 63°C for 10 min. After incubation at room temperature for 5 min, the resuspended library was transferred to a fresh PCR tube and 30μL of Hybridization Enhancer was added to the top of the library. For hybridization of the probes, the sample was incubated at 95°C for 30 s followed by 60°C for ∼16 h in a T100 thermal cycler (Bio-Rad). Next, 100 μL of Dynabeads M-270 Streptavidin (Thermo Fisher Scientific) was added to a fresh 1.5 mL tube and washed three times with 200 μL of Fast Binding Buffer using a magnetic stand DynaMag-2 (Thermo Fisher Scientific). After washing, the bead pellet was resuspended in 200 μL of Fast Binding Buffer and the suspension was incubated for >10 min at 63°C in a heat block. After the hybridization reaction, the sample was added to the incubated bead suspension without removing the PCR tube from the thermal cycler. After incubation for 5 min at 63°C, the sample was placed on a magnetic stand and the supernatant was removed. The bead pellet was suspended in 200 μL of Fast Wash Buffer 1 preheated at 63°C. After incubation for 5 min at 63°C, the tube was placed on a magnetic stand and the suspension was removed. The bead pellet was suspended in 200 μL of preheated Fast Wash Buffer 1. After incubation for 5 min at 63°C, the suspension was transferred to a fresh 1.5-mL tube. The tube was set on a magnetic stand and the suspension was removed. The bead pellet was resuspended in 200 μL of Wash Buffer 2 and preheated at 48°C. After incubation for 5 min at 48°C, the tube was placed on a magnetic stand and the suspension was removed. A wash step using Wash Buffer 2 was performed two more times (three times in total). Elution, amplification, sequencing, and data analysis of the captured library were performed in the same manner as the final version of t-nanoEM using the Twist Standard Hyb and Wash Kit v2.

#### Evaluation of PCR enzymes

1 ng of the nanoEM v2 library was used as a template. Each PCR reaction was prepared at 50 μL scale following the manufacturer’s protocol with 1.5 μL of Amplification Primers (Twist Bioscience). The PCR reaction was conducted under the following conditions: 1 cycle of 1 min at 94°C, 7 cycles of 15 s at 94°C, 30 s at 58.8°C, and 15 min at 68°C, 1 cycle of 15 min at 68°C for KOD FX (TOYOBO); 7 cycles of 15 s at 94°C, 5 s at 58.8°C, and 15 min at 68°C for KOD ONE (TOYOBO); 1 cycle of 1 min at 94°C, 7 cycles of 15 s at 94°C, 30 s at 63.8°C, and 15 min at 68°C, 1 cycle of 15 min at 68°C for KOD FX *Neo* (TOYOBO). The amplified libraries were purified and eluted in 20 μL of NFW using a DNA Clean & Concentrator-5 (Zymo Research). The purified libraries were quantified using a DNA 12000 Kit and a 2100 Bioanalyzer (Agilent Technologies).

#### Examination of t-nanoEM from FFPE samples

Genomic DNA was extracted from FFPE sections of mouse kidneys using the CELLDATA DNAstorm 2.0 FFPE DNA Extraction Kit (Biotium). 50 ng of the extracted DNA was used for library preparation for nanoEM, except for the final size-selection step. To evaluate the distribution of the DNA fragment length and quantitation, a DNA12000 kit with a 2100 Bioanalyzer (Agilent Technologies) and a Qubit dsDNA HS Assay Kit with a Qubit 4 Fluorometer (Thermo Fisher Scientific) were used, respectively.

### Quantification and statistical analysis

Details of the specific statistical tests and software are described in the figure legends or within the STAR Methods. In this study, the term “average” refers to the mean.

## Supplemental information

Document S1. Figures S1–S5 and Table S3

Table S1. Summary of statistics in this study, related to Figures 2–5

Table S2. Lists of DMRs obtained in this study, related to Figures 2–5

Data S1. Bed files of target regions for the custom panels, related to Figures 3–5

Document S2. Article plus supplemental information
