# Supplementary material for: Targeted long-read methylation analysis using hybridization capture suitable for clinical specimens
Source: Cell Rep Methods. 2025 Nov 3;5(11):101215. doi: 10.1016/j.crmeth.2025.101215 (PMC12664885; doi:10.1016/j.crmeth.2025.101215)
Supplement: Document S1. Figures S1–S5 and Table S3 [file mmc1.pdf]

Cell Reports Methods, Volume *5*

Supplemental information

Targeted long-read methylation analysis using hybridization capture suitable for clinical specimens

Keisuke Kunigo, Satoi Nagasawa, Keiko Kajiya, Yoshitaka Sakamoto, Suzuko Zaha, Yuta Kuze, Akinori Kanai, Kotaro Nomura, Masahiro Tsuboi, Genichiro Ishii, Ai Motoyoshi, Koichiro Tsugawa, Motohiro Chosokabe, Junki Koike, Ayako Suzuki, Yutaka Suzuki, and Masahide Seki

Table S3. PCR primers used to prepare libraries for nanoEM v2, related to STAR METHODS.

	i7 primer	i5 primer	i7 index	i5 index
Index1	CAAGCAGAAGACGGCATACGAGATCGAGTAATGTGACTGGAGTTCAGACGTGTGCTCTTCCGATCT	AATGATACGGCGACCACCGAGATCTACACTATAGCCTACACTCTTTCCCTACACGACGCTCTTCCGATCT	CGAGTAAT	TATAGCCT
Index2	CAAGCAGAAGACGGCATACGAGATTCTCCGGAGTGACTGGAGTTCAGACGTGTGCTCTTCCGATCT	AATGATACGGCGACCACCGAGATCTACACATAGAGGCACACTCTTTCCCTACACGACGCTCTTCCGATCT	TCTCCGGA	ATAGAGGC
Index3	CAAGCAGAAGACGGCATACGAGATAATGAGCGGTGACTGGAGTTCAGACGTGTGCTCTTCCGATCT	AATGATACGGCGACCACCGAGATCTACACCCTATCCTACACTCTTTCCCTACACGACGCTCTTCCGATCT	AATGAGCG	CCTATCCT
Index4	CAAGCAGAAGACGGCATACGAGATGGAATCTCGTGACTGGAGTTCAGACGTGTGCTCTTCCGATCT	AATGATACGGCGACCACCGAGATCTACACGGCTCTGAACACTCTTTCCCTACACGACGCTCTTCCGATCT	GGAATCTC	GGCTCTGA
Index5	CAAGCAGAAGACGGCATACGAGATTTCTGAATGTGACTGGAGTTCAGACGTGTGCTCTTCCGATCT	AATGATACGGCGACCACCGAGATCTACACAGGCGAAGACACTCTTTCCCTACACGACGCTCTTCCGATCT	TTCTGAAT	AGGCGAAG

A

Method	Input amount	Num of target regions or target size	DNA methylation detection
PCR	Low (e.g. 1 ng[13])	Low (e.g. 36 regions[s4])	Possible by combining with base conversion[13-15]
Hybrid capture	Medium (e.g. 200 ng*)	Quite high (e.g. 4,800 genes[s5])	**Not applicable**
Cas9-mediated	High (e.g. 3 μg[9])	Low (e.g. 10 regions[9])	Possible by basecalling of modified bases[9]
Adaptive sampling	High (e.g. 2 μg**)	Quite high (e.g. 310 Mb**)	Possible by basecalling of modified bases**

*https://www.twistbioscience.com/resources/protocol/long-read-library-preparation-and-standard-hyb-v2-enrichment
**https://nanoporetech.com/document/reduced-representation-methylation-multiplex-sequencing-v14

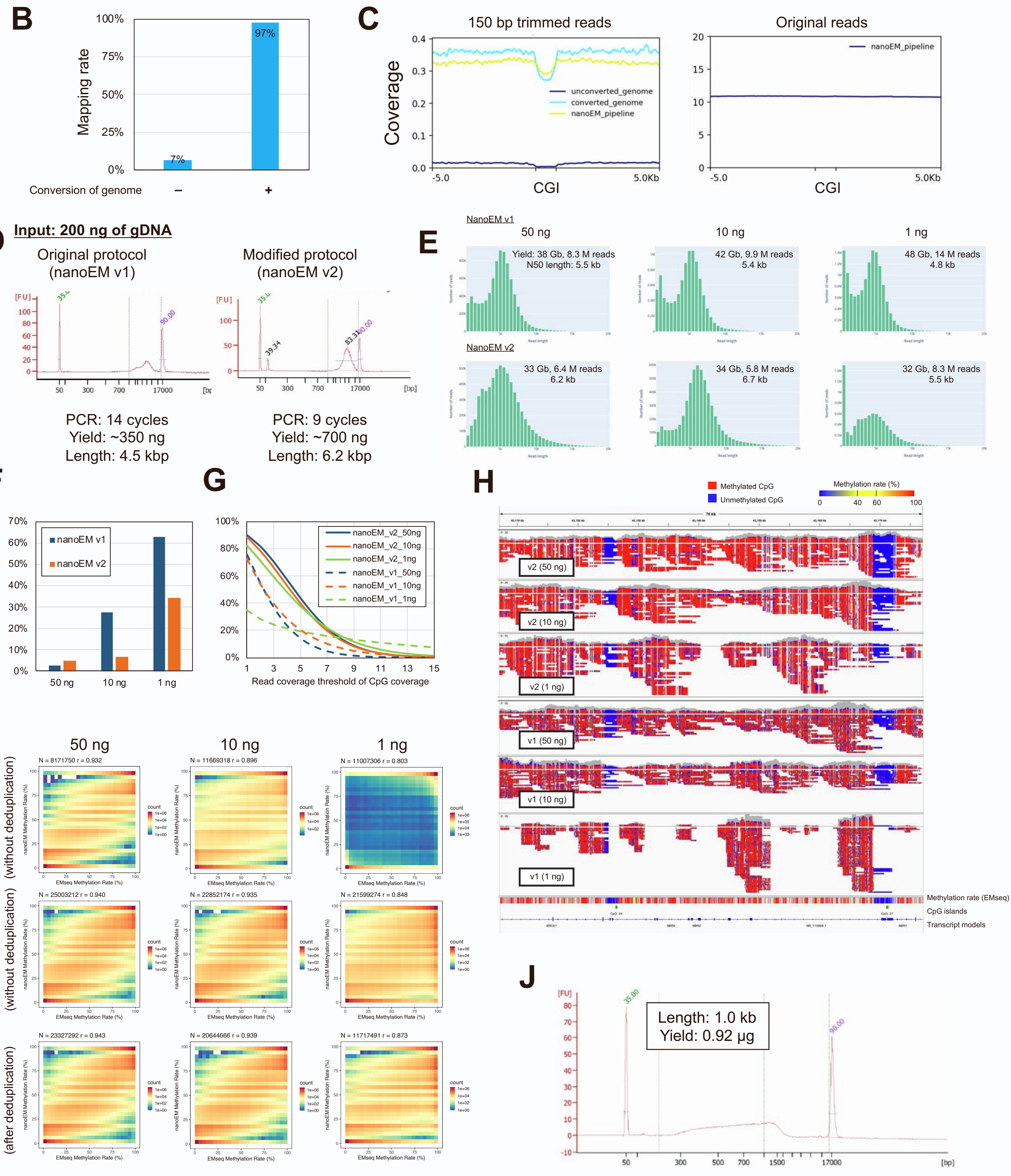

Figure S1. Investigation of methods for target sequencing methods and optimization of nanoEM, related to Figure 1.

(**A**) **Pros and cons of targeted long-read sequencing methods.**

(**B** and **C**) **Simulation of nanoEM application to adaptive sampling.**

We conducted a simulation using whole-genome nanoEM v2 data obtained from 50 ng of MB231 gDNA. In our experience, the average sequence length used for adaptive sampling is 150 bp. Therefore, we extracted the first 150 bp of each nanoEM v2 read following adapter trimming. (**B**) shows mapping rates for the 150 trimmed reads aligned to two different reference genomes: the standard reference genome and the converted genome (combining a C-to-T substituted genome and a G-to-A substituted genome). (**C**) shows coverage depth of the trimmed and untrimmed original reads around the CpG islands (CGIs). Trimmed reads were aligned to the standard and the converted reference genome. Trimmed and original reads processed by the nanoEM pipeline are also shown. Although only ~7% of the trimmed reads were aligned to the standard unconverted genome, 97% of the reads were aligned to the converted genome (**B**); however, the coverage within CGIs was considerably lower compared with that of the untrimmed original reads aligned using our nanoEM pipeline (**C**). In the nanoEM pipeline, methylated Cs (or its complementary Gs) on reads are *in silico* substituted with Ts (or As), and then mapped to the converted genome. Although we observed a slight improvement in this tendency using the nanoEM pipeline, the reduced coverage within the CGIs persisted. This suggests that aligning short reads to CGIs, which are important targets for DNA methylation regulation, is a significant challenge. Consequently, adaptive sampling may result in less efficient capture of reads derived from CGIs compared with hybridization capture methods. Therefore, based on these observations, effectively applying nanoEM to adaptive sampling would be challenging.

(**D–I**) **Optimization of the reaction conditions for whole-genome nanoEM.**

(**D**) Measurements of whole-genome nanoEM libraries prepared from 200 ng of MB231 gDNA extracted from MB231 using the original protocol (nanoEM v1)[81] and the modified protocol (nanoEM v2). The libraries without size-selection were quantified using a DNA 12000 Kit with a 2100 Bioanalyzer (Agilent Technologies). (**E**) Length distributions of the 1d pass read of nanoEM v1 and v2 prepared from 50, 10, and 1 ng of MB231 gDNA. The datasets for nanoEM v1 and v2 were obtained from our previous study[5] and this study, respectively. The total sequenced bases (Gb), the number, and the N50 length of the 1d pass reads are shown in the plots. In nanoEM v2, the N50 lengths of the reads were improved, although the yields of the sequencing data in v2 were somewhat lower compared with those in v1. Because the R9.4.1 flow cell tends to show a higher yield of sequencing compared with R10 and later flow cells[S1], this difference is due to the different versions of the PromethION flow cells used for v1 and v2 (R9.4.1 and R10.4.1, respectively). (**F**) Duplicate rates of nanoEM v1 and v2 prepared from 50, 10, and 1 ng of gDNA extracted from MB231 cells. The duplication rate was estimated using a script (https://github.com/yos-sk/nanoEM/blob/master/analysis/detect_PCR_duplicates.py) used in our previous study[5]. In this script, the 5′ and 3′ positions were clustered with a 10-bp margin and reads belonging to each cluster, except for one read, were judged as PCR duplicates because even PCR duplicates could shift the position of the mapping ends by a few bases between reads generated by R9.4 flow cells. (**G**) The distribution of CpG coverage in nanoEM v1 and v2 without the removal of the PCR duplicates. The relationship between the percent covered CpG and the read coverage threshold of CpG are shown. (**H**) Typical examples of the distribution of nanoEM v1 and v2 reads for each input amount around the BRCA1 locus. The mapped reads without the removal of PCR duplicates are displayed in the IGV. Read coverages and distributions in nanoEM v2 and v1 are shown in the top panel. The methylation rate measured by short-read EM-seq, CpG islands, and RefSeq transcript models is shown in the bottom panel. (**I**) Scatter plots of the methylation rate of CpG covered by five reads or more between short-read EM-seq and nanoEM v1 or v2. For the nanoEM v1, PCR duplicates were not removed (upper panels). For the nanoEM v2 data, we showed both the data before and after removal of PCR duplicates (middle and lower panels, respectively). PCR duplicates were removed using MarkDuplicates of Picard. The frequency of the dot counts within each bin is displayed in a heatmap. The frequency of the dot counts within each bin is displayed in a heatmap.

(**J**) **NanoEM v2 library prepared from the FFPE section.**

(**J**) shows quantification result for the nanoEM library prepared from FFPE sections of mouse liver. Its length was only ~1kb, because genomic DNA extracted from FFPE sections is fragmented and contains nicks. Therefore, we used fresh frozen (FF) sections, but not FFPE sections for this study.

A

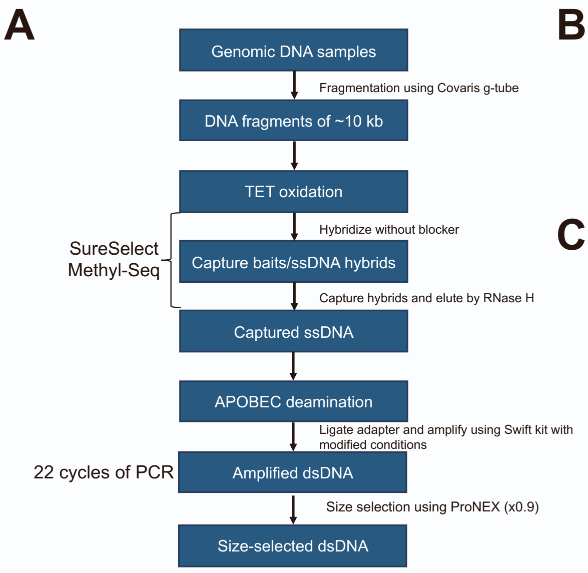

B

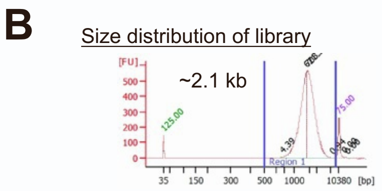

C

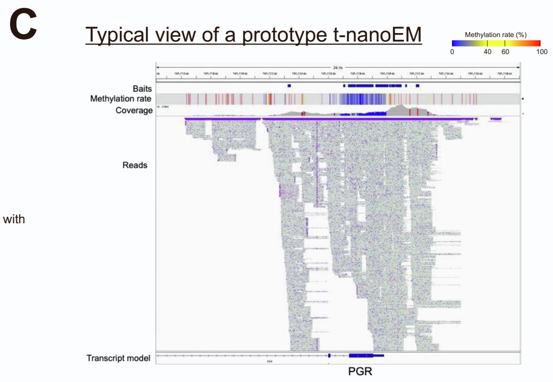

D

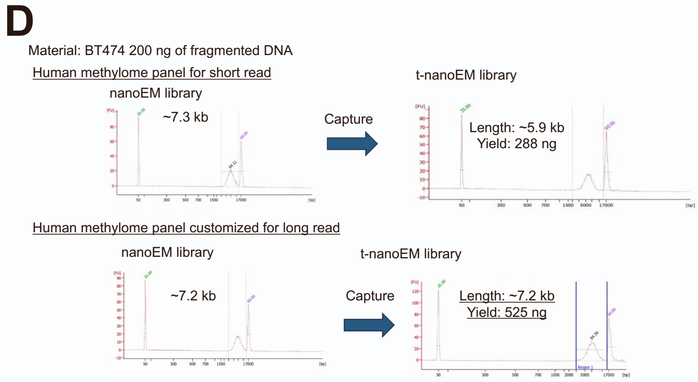

E

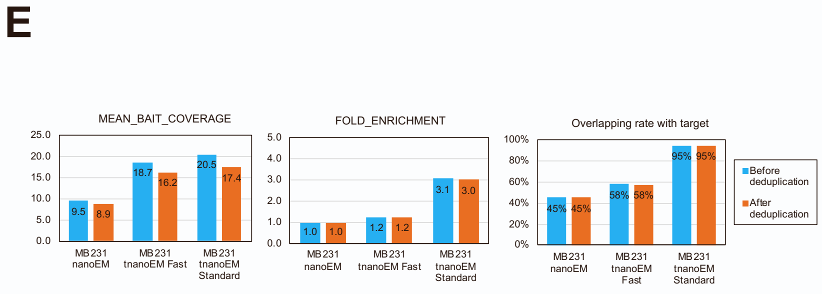

F

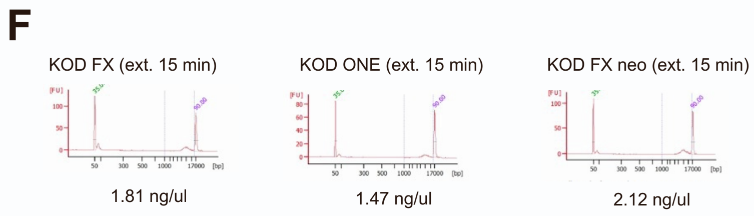

G

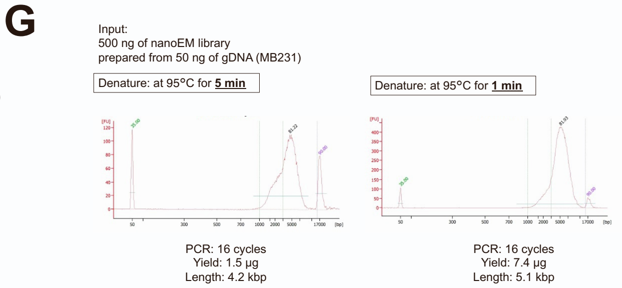

H

#substitution patterns	Forward reads (with C->T conversion)				Reverse reads (with g->a conversion)			
	A	T	G	C	a (T)	t (A)	g (C)	c (G)
C->T	-	-	-	ref	-	alt	-	ref
C->A	alt	ref	-	ref	alt	-	-	ref
C->G	-	ref	alt	ref	alt	-	alt	ref
A->T	ref	alt	-	-	ref	alt	-	-
A->G	ref	-	alt	-	-	-	alt	-
A->C	ref	alt	-	alt	ref	-	-	alt
G->A	alt	-	ref	-	-	-	ref	-
G->T	-	alt	ref	-	ref	alt	ref	-
G->C	-	alt	ref	alt	ref	-	ref	alt
T->A	alt	ref	-	-	alt	ref	-	-
T->G	-	ref	alt	-	alt	ref	alt	-
T->C	-	-	-	alt	-	ref	-	alt

I

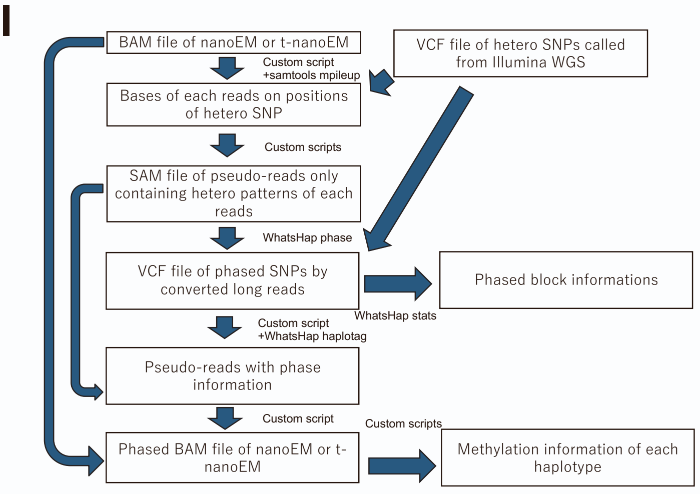

Figure S2. Optimization of targeted nanoEM, related to Figure 1.
(**A–C**) **Prototype of t-nanoEM using the SureSelectXT Methyl-Seq system.**
Workflow (**A**), size distribution of the library (**B**), and typical view (**C**) of a prototype version of t-nanoEM using the SureSelectXT Methyl-Seq system (Agilent Technologies) (for the detailed protocol, see **STAR Methods**). (**C**) Typical views of the distribution of mapped reads after deduplication are displayed in IGV. Target regions and the CpG methylation rate measured by the prototype t-nanoEM are shown in the top panel. Read coverages and distribution of the prototype t-nanoEM of BT474 and RefSeq transcript models are shown in the bottom panel. Before this version, we examined the capture of a long fragment (<10 kb) with adapter ligation before hybridization capture using SureSelect Methyl-Seq. Because we could not obtain any amplicons by PCR after capture, even without base conversion in the trial, we employed the adapter ligation method of Swift Biosciences following target capture; however, the length of the library prepared by this protocol was only ~2.1 kb and a high duplication rate was observed. In our subsequent study, we found that >5 kb of DNA could be captured without base conversion (https://www.agilent.com/cs/library/applications/an-long-read-sureselect-xt-hs2-5994-7612en-agilent.pdf), but we did not use the SureSelectXT system in this study because it requires >500 ng of gDNA input. Therefore, we used a target enrichment system after base conversion and PCR released from Twist Bioscience.
(**D**) **Optimization of the capture panel for t-nanoEM.**
The size distributions of the t-nanoEM and nanoEM libraries which is the sources of the t-nanoEM libraries are shown. The upper panel shows the results of target capture when using the ready-made human methylome panel for short-read sequencing. The lower panel shows the results when using the custom human methylome panel in which probes were more sparsely tiled on the target regions compared with the panel for short-read sequencing. A total of 200 ng of nanoEM v2 libraries were captured using the ready-made human methylome panel or the custom human methylome panel with the Fast Hybridization and Wash Kit (for the detailed protocol, see **STAR Methods**). The size distribution and quantity of the libraries were measured using a DNA12000 kit with a 2100 Bioanalyzer (Agilent Technologies) and a Qubit dsDNA HS Assay Kit with a Qubit 4 Fluorometer (Thermo Fisher Scientific), respectively. The library prepared with the custom human methylome panel showed a longer length and higher yield. Therefore, we employed the custom human methylome panel.
(**E**) **Comparison of a prototype and the final version of t-nanoEM using the Twist system.**
Comparison of the protype version of t-nanoEM using the Twist Fast Hybridization and Wash Kit (t-nanoEM Fast) with the whole-genome nanoEM v2 and the final version of t-nanoEM using the Twist Standard Hyb and Wash Kit v2 (t-nanoEM Standard) in the target enrichment metrics. All libraries were prepared from 10 ng of MB231 gDNA. Although the t-nanoEM Fast showed comparable mean bait coverage to the t-nanoEM Standard, this was simply because the yield of reads in Fast was higher than that of Standard (8,497,684 and 5,770,107 reads for Fast and Standard, respectively). Regarding the fold-enrichment and overlapping rate with the target regions, which reflect the efficiency of the target capture, the t-nanoEM Standard exhibited superior performance. Therefore, we used the protocol with the Twist Standard Hyb and Wash Kit v2 (see **STAR Methods** section for the detailed protocol).
(**F**) **Optimization of postcapture PCR conditions.**
Using a nanoEM v2 library as a mock sample after target capture, we compared KOD FX (equivalent to KOD Xtreme Hot Start DNA Polymerase), KOD FX Neo, and KOD ONE (TOYOBO). KOD FX Neo showed the highest library yield (for the detailed protocol, see **STAR Methods**). Therefore, we used KOD FX Neo for postcapture PCR.
(**G**) **Optimization of the denaturing conditions prior to hybridization.**
The denaturation time for hybridization capture was compared between 5 min (the original condition) and 1 min. Using 500 ng of nanoEM v2 libraries prepared from 50 ng of gDNA, library preparation for t-nanoEM with the Twist Standard Hyb and Wash Kit v2 and the custom human methylome panel was performed. The prepared libraries without size-selection were quantified using a DNA12000 kit with an 2100 Bioanalyzer. The yield and the length of the t-nanoEM library were greatly improved by shortening the denaturation time. Therefore, we used these denaturation conditions. Because too much of the library was amplified under these conditions, the number of PCR cycles was reduced to 12 in the final protocol when using the custom human methylome panel.
(**H**) **Discrimination of base substitution patterns considering base conversion.**
(**I**) **A workflow for haplotype phasing using converted long-reads.**

An overview of the workflow for hetero SNP phasing and methylation phasing using nanoEM or t-nanoEM reads is shown. For a detailed explanation, see the **STAR Methods** and the website (https://github.com/masahide-seki/t-nanoEM). Scripts for these analyses are available on the website.

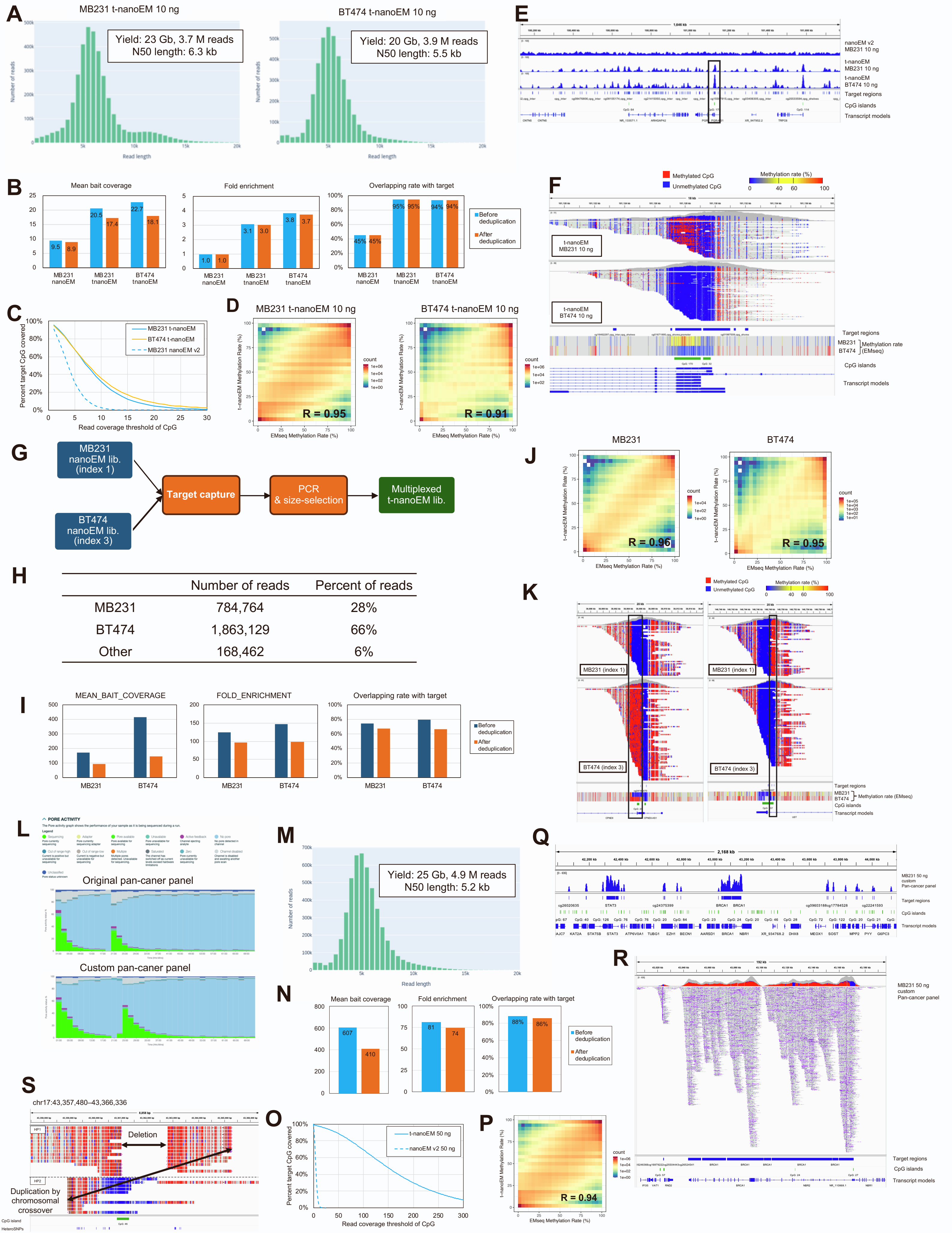
A
MB231 t-nanoEM 10 ng
Yield: 23 Gb, 3.7 M reads
N50 length: 6.3 kb
BT474 t-nanoEM 10 ng
Yield: 20 Gb, 3.9 M reads
N50 length: 5.5 kb
Number of reads
Read length
E
nanoEM v2 MB231 10 ng
t-nanoEM MB231 10 ng
t-nanoEM BT474 10 ng
Target regions
CpG islands
Transcript models
B
Mean bait coverage
Fold enrichment
Overlapping rate with target
Before deduplication
After deduplication
MB231 nanoEM
MB231 tnanoEM
BT474 tnanoEM
9.5
8.9
20.5
17.4
22.7
18.1
1.0
1.0
3.1
3.0
3.8
3.7
45%
45%
95%
95%
94%
94%
F
Methylated CpG
Unmethylated CpG
Methylation rate (%)
t-nanoEM MB231 10 ng
t-nanoEM BT474 10 ng
Target regions
MB231
BT474
Methylation rate (EMseq)
CpG islands
Transcript models
C
Percent target CpG covered
Read coverage threshold of CpG
MB231 t-nanoEM
BT474 t-nanoEM
MB231 nanoEM v2
D
MB231 t-nanoEM 10 ng
BT474 t-nanoEM 10 ng
t-nanoEM Methylation Rate (%)
EMseq Methylation Rate (%)
count
R = 0.95
R = 0.91
G
MB231 nanoEM lib. (index 1)
BT474 nanoEM lib. (index 3)
Target capture
PCR & size-selection
Multiplexed t-nanoEM lib.
J
MB231
BT474
R = 0.96
R = 0.95
H
Number of reads | Percent of reads
MB231 | 784,764 | 28%
BT474 | 1,863,129 | 66%
Other | 168,462 | 6%
K
Methylated CpG
Unmethylated CpG
Methylation rate (%)
MB231 (index 1)
BT474 (index 3)
Target regions
MB231
BT474
Methylation rate (EMseq)
CpG islands
Transcript models
I
MEAN_BAIT_COVERAGE
FOLD_ENRICHMENT
Overlapping rate with target
Before deduplication
After deduplication
MB231
BT474
L
PORE ACTIVITY
Original pan-caner panel
Custom pan-caner panel
M
Yield: 25 Gb, 4.9 M reads
N50 length: 5.2 kb
Number of reads
Read length
Q
MB231 50 ng custom Pan-cancer panel
Target regions
CpG islands
Transcript models
R
MB231 50 ng custom Pan-cancer panel
Target regions
CpG islands
Transcript models
N
Mean bait coverage
Fold enrichment
Overlapping rate with target
607
410
81
74
88%
86%
Before deduplication
After deduplication
S
chr17:43,357,480–43,366,336
HP1
HP2
Deletion
Duplication by chromosomal crossover
CpG island
HeteroSNPs
O
Percent target CpG covered
Read coverage threshold of CpG
t-nanoEM 50 ng
nanoEM v2 50 ng
P
t-nanoEM Methylation Rate (%)
EMseq Methylation Rate (%)
count
R = 0.94

Figure S3. The results of t-nanoEM using the cell lines, related to Figure 2.
(**A**–**F**) **Assessment of t-nanoEM prepared with the custom human methylome panel.**
The results and evaluation of t-nanoEM prepared from 10 ng of gDNA extracted from MB231 and BT474 cells with the custom human methylome panel. (**A**) Length distribution of the 1d pass read of t-nanoEM. The total sequenced bases (Gb), the number, and the N50 length of the t-nanoEM reads are shown in squares. (**B**) Mean bait coverage, fold-enrichment, and overlapping rate before and after deduplication of t-nanoEM and nanoEM v2 prepared from 10 ng of MB231 gDNA and t-nanoEM from 10 ng of BT474 gDNA are shown. (**C**) The percentage of covered CpG by the read coverage threshold of CpG by t-nanoEM and whole-genome nanoEM v2 after deduplication is shown. (**D**) Scatter plots of the methylation rate of CpG covered by five reads or more between short-read EM-seq and t-nanoEM after deduplication. The frequency of dot counts within each bin is displayed as a heatmap. (**E** and **F**) Typical views of the distribution of mapped reads after deduplication are displayed in IGV. (**E**) Read coverage in nanoEM v2 and t-nanoEM is shown in the top panel. The target regions, CpG islands, and RefSeq transcript models are shown in the bottom panel. (**F**) Enlargement of the area enclosed by the square in **E**. In the top panel, the read coverage and read distribution of t-nanoEM are shown by the bisulfite mode of IGV, in which methylated and unmethylated CpGs are shown in red and blue, respectively. Target regions, CpG islands, CpG methylation rate measured by short-read EM-seq in our previous study[5], and RefSeq transcript models are shown in the bottom panel.
(**G**–**K**) **Examination of pre-pooling of libraries before capture.**
(**G**) Schematic view of the workflow of pre-pooling. (**H**) The number and percentage of multiplexed t-nanoEM prepared from 10 ng each of gDNA from MB231 and BT474 cells with the pan-cancer panel after demultiplexing. (**I**) Mean bait coverage, fold-enrichment, and overlapping rate before and after deduplication of the demultiplexed t-nanoEM are shown. (**J**) Scatter plots of the methylation rate of CpG covered by five reads or more between short-read EM-seq and t-nanoEM after deduplication. (**K**) Typical views of the distribution of mapped reads after deduplication are displayed in the IGV. Read coverages in the demultiplexed t-nanoEM of MB231 and BT474 cells are shown in the top panel. The target regions, CpG methylation rate measured by short-read EM-seq, CpG islands, and RefSeq transcript models are shown in the bottom panel.
(**L**–**R**) **Assessment of t-nanoEM prepared using the custom pan-cancer panel.**
The results of t-nanoEM prepared from 50 ng of MB231 gDNA with a custom pan-cancer panel. (**L**) Pore activity of t-nanoEM using the original and custom pan-cancer panels. Graphs for the pore activities during nanopore sequencing for the original and custom pan-cancer panels. (**M**) Length distributions for the t-nanoEM 1d pass reads. The total sequenced bases (Gb), number, and N50 length of the t-nanoEM reads are shown in squares. (**N**) Mean bait coverage, fold-enrichment, and overlapping rate before and after deduplication of t-nanoEM and nanoEM v2 prepared from the same amount of gDNA as t-nanoEM. (**O**) The percentage of CpG covered by the read coverage threshold of CpG by t-nanoEM and nanoEM v2 following deduplication. (**P**) Scatter plots for the methylation rate of CpG covered by five reads or more between short-read EM-seq and t-nanoEM following deduplication. (**Q** and **R**) Typical views of the distribution of mapped reads following deduplication are displayed in the IGV. The read coverage in nanoEM v2 and t-nanoEM is shown in the top panel. The target regions, CpG islands, and RefSeq transcript models are shown in the bottom panel. (**R**) Enlargement of the area enclosed by the square in **Q**. The read coverage and read distribution of t-nanoEM are shown by the bisulfite mode of IGV in the top panel, in which methylated and unmethylated CpGs are shown in red and blue, respectively. The target regions, CpG islands, and RefSeq transcript models are shown in the bottom panel.
The yield of t-nanoEM using the pan-cancer panel (14 Gb) is lower compared with typical flowcell yields (**Table S1A**). This is primarily due to rapid pore inactivation (**L**). For example, the nanopore WGS data in the present study was 73 Gb (**Table S1J**). However, when considering the yields per flowcell for the whole-genome nanoEM v2 (32–34 Gb), those of t-nanoEM with the panel were not remarkably low. This suggests that the sequencing of EM-seq-converted libraries is less efficient compared with that of nonconverted libraries. Despite the lower yield, the fold enrichment rates for the panel (x131–175) enabled us to obtain a higher depth of the target regions (**Figures 2B** and **5B**). The t-nanoEM data obtained using the custom human methylome panel showed higher yields (20–37 Gb) compared with those obtained from the pan-cancer panel (**Table S1A**). This may be attributed to probe design, where probes for long-reads (including those for the custom methylome panel) are tiled more sparingly compared with those for short reads (including those for the pan-cancer panel). We designed a new custom pan-cancer panel that targets regions from the original panel along with additional cancer-related

genes to improve the yield. The custom Pan-cancer panel demonstrated improved pore activity and yield (25 Gb) with a performance comparable to that of the original panel.

(S) Methylation status of a complex SNV detected by t-nanoEM.

An example of a complex SV detected in the t-nanoEM data. The reads for the t-nanoEM prepared from 50 ng of BT474 gDNA using the custom human methylome panel covering a complex SV are shown. We observed a complex SV, which is a combination of a deletion and a chromosomal crossover between haplotypes. Interestingly, while haplotype 2 harboring the deletion was unmethylated, haplotype 1 with deletion was highly methylated. This difference may be attributed to the deletion of a CpG island in haplotype 1.

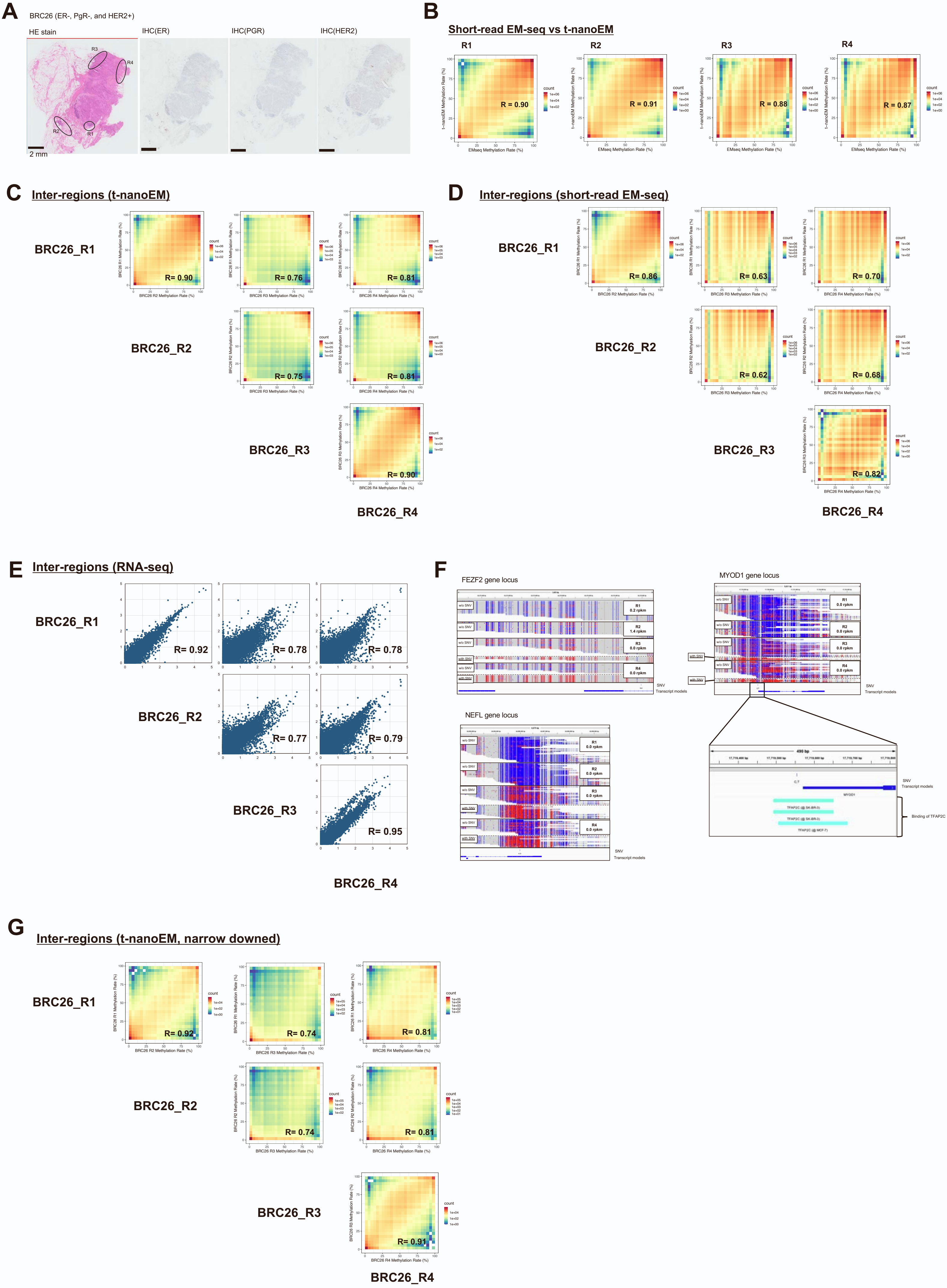
A
BRC26 (ER-, PgR-, and HER2+)
HE stain
IHC(ER)
IHC(PGR)
IHC(HER2)
R1
R2
R3
R4
2 mm
B
Short-read EM-seq vs t-nanoEM
R1
R2
R3
R4
R = 0.90
R = 0.91
R = 0.88
R = 0.87
t-nanoEM Methylation Rate (%)
EMseq Methylation Rate (%)
count
C
Inter-regions (t-nanoEM)
BRC26_R1
BRC26_R2
BRC26_R3
BRC26_R4
R= 0.90
R= 0.76
R= 0.81
R= 0.75
R= 0.81
R= 0.90
D
Inter-regions (short-read EM-seq)
BRC26_R1
BRC26_R2
BRC26_R3
BRC26_R4
R= 0.86
R= 0.63
R= 0.70
R= 0.62
R= 0.68
R= 0.82
E
Inter-regions (RNA-seq)
BRC26_R1
BRC26_R2
BRC26_R3
BRC26_R4
R= 0.92
R= 0.78
R= 0.78
R= 0.77
R= 0.79
R= 0.95
F
FEZF2 gene locus
MYOD1 gene locus
NEFL gene locus
w/o SNV
with SNV
R1 0.2 rpkm
R2 1.4 rpkm
R3 0.0 rpkm
R4 0.0 rpkm
R1 0.0 rpkm
R2 0.0 rpkm
R3 0.0 rpkm
R4 0.0 rpkm
SNV
Transcript models
490 bp
MYOD1
Binding of TFAP2C
G
Inter-regions (t-nanoEM, narrow downed)
BRC26_R1
BRC26_R2
BRC26_R3
BRC26_R4
R= 0.92
R= 0.74
R= 0.81
R= 0.74
R= 0.81
R= 0.91

Figure S4. The results of t-nanoEM using the breast cancer specimen, related to Figure 4.
(**A**) **HE staining and immunohistochemistry of a breast cancer specimen.**
HE staining and immunohistochemistry (IHC) of ER, PGR, and HER2 in BRC26 cells are shown. The BRC26 specimen was surgically removed from a patient who was not undergoing chemotherapy. From IHC and FISH (fluorescent *in situ* hybridization), the specimen was classified as HER2-positive, although the staining was somewhat unclear because of the nature of the frozen section. HE staining, IHC, and FISH were performed as described in our previous study[32].
(**B**) **Correlation of the CpG methylation rates of short-read EM-seq and t-nanoEM in each region dissected from breast cancer tissue samples.**
CpG methylation rates were compared between t-nanoEM and short-read EM-seq prepared from the same gDNA of the microdissected breast cancer tissues. Scatter plots of the CpG methylation rates covered by five reads or more between short-read EM-seq and t-nanoEM after deduplication. The frequency of the dot counts within each bin is displayed as a heatmap. Pearson's correlation coefficients are shown for each plot.
(**C**–**E**) **Inter-sample correlation analysis among microdissected breast cancer tissue samples using t-nanoEM, short-read EM-seq, and RNA-seq.**
CpG methylation rates measured by t-nanoEM (**C**) and EM-seq (**D**) and gene expression levels measured by RNA-seq (**E**) were compared among microdissected breast cancer tissue samples. For methylation data, scatter plots were generated to show the correlation between methylation rates (for CpGs covered by five reads or more after deduplication). For gene expression, Pearson's correlation coefficients were calculated for log10 (rpkm + 1) values of genes expressed at least 1 rpkm.
(**F**) **Methylation status of the allele with SNV around tumor suppressor genes.**
Examples of DMRs on alleles with SNVs around three tumor suppressor genes, FEZF2, MYOD1, and NEFL genes. For each sample, the reads assigned with and without SNVs and the CpG methylation status are visualized in the bisulfite mode of IGV in the top panel. Regarding R1 and R2, SNV reads were not detected at these loci. SNVs and the RefSeq transcript models are shown in the bottom panel. For MYOD1, an enlarged view around the SNV is shown. In the bottom of the panel, the binding sites of the transcription factor TFAP2C were visualized using the peak browser function of ChIP-Atlas[S2].
The SNV of the MYOD1 gene locus was located in a binding site of the transcription factor TFAP2C, which is a negative regulator of the Myod1 gene in mice[S3]. It is possible that the SNV altered the binding affinity of TFAP2C, resulting in increased methylation levels in the MYOD1 gene.
(**G**) **Inter-sample correlation analysis of CpG methylation rates among breast cancer tissues measured by t-nanoEM when narrowed down to pan-cancer panel targets.**
CpG methylation rates were compared among the t-nanoEM of the microdissected breast cancer tissues. The reads overlapping with the target regions of the pan-cancer panel were extracted from the reads of t-nanoEM captured by the custom human methylome panel. Scatter plots of the CpG methylation rates covered by five or more reads in both regions. The frequency of the dot counts within each bin is shown as a heatmap. Each plot shows Pearson's correlation coefficients.
98% of the target regions in the pan-cancer panel overlapped with the custom human methylome panel. To assess the performance differences between these panels, t-nanoEM reads were extracted from breast cancer tissues that overlapped with the pan-cancer panel, and the correlation coefficients from the extracted data were estimated. Compared with the original data (**C**), the correlation coefficients were slightly higher within nontumor and tumor areas, whereas they were slightly lower between the nontumor and tumor areas. This indicates that the pan-cancer panel includes a higher proportion of target regions that characterize the differences between nontumor and tumor tissues compared with that in the custom human methylome panel.

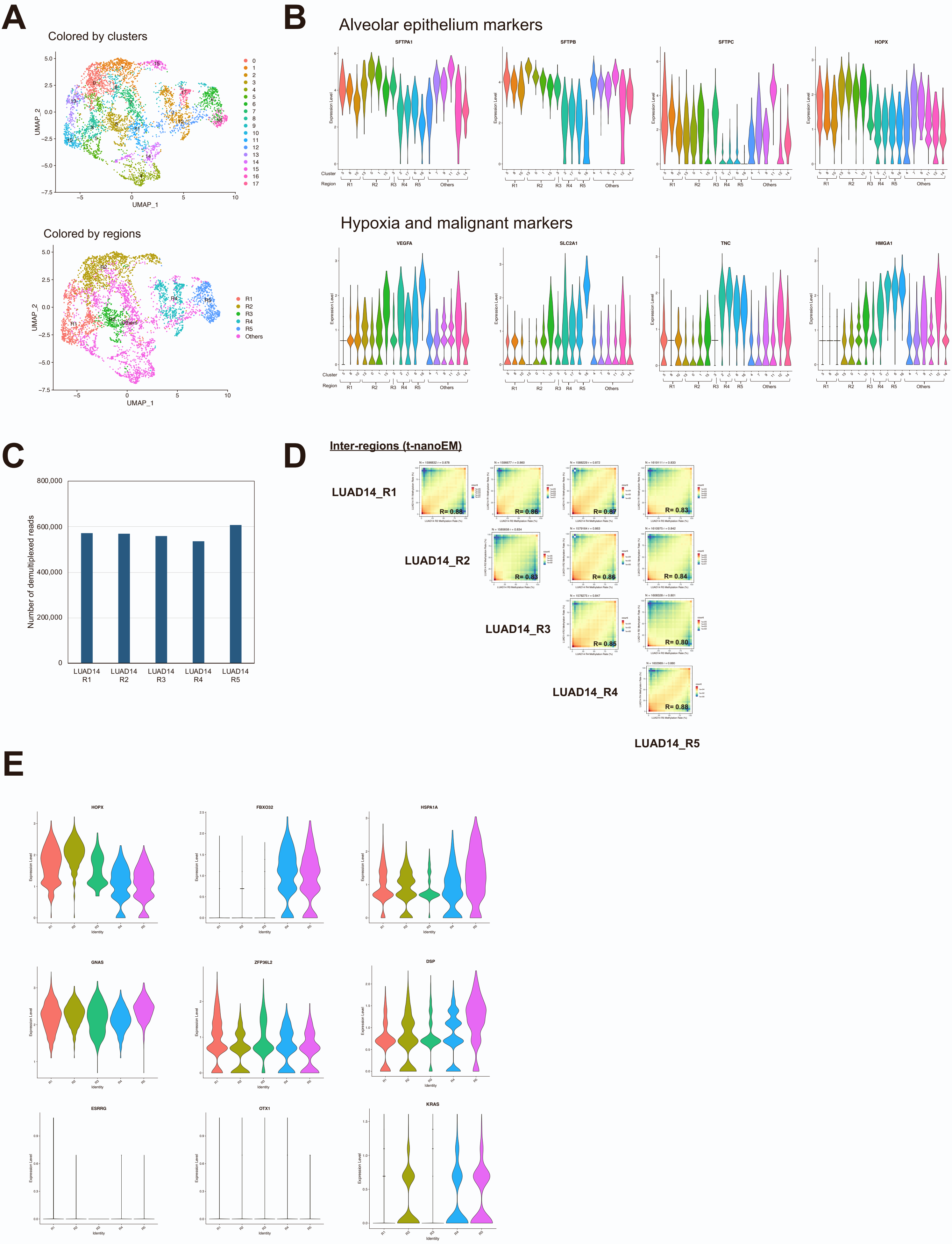

A
Colored by clusters
UMAP_1
UMAP_2
Colored by regions
R1
R2
R3
R4
R5
Others
B
Alveolar epithelium markers
SFTPA1
SFTPB
SFTPC
HOPX
Hypoxia and malignant markers
VEGFA
SLC2A1
TNC
HMGA1
Cluster
Region
Expression Level
C
Number of demultiplexed reads
800,000
600,000
400,000
200,000
0
LUAD14 R1
LUAD14 R2
LUAD14 R3
LUAD14 R4
LUAD14 R5
D
Inter-regions (t-nanoEM)
LUAD14_R1
LUAD14_R2
LUAD14_R3
LUAD14_R4
LUAD14_R5
R= 0.88
R= 0.86
R= 0.87
R= 0.83
R= 0.83
R= 0.86
R= 0.84
R= 0.85
R= 0.80
R= 0.88
E
HOPX
FBXO32
HSPA1A
GNAS
ZFP36L2
DSP
ESRRG
OTX1
KRAS
Identity

Figure S5. The results of t-nanoEM using the breast cancer specimen, related to Figure 5.
(**A**) **Clusters and expression levels of marker genes in the Visium data.**
UMAP plots colored by clusters and regions are shown in the upper and lower panels, respectively. UMAP plots were generated by the DimPlot function of Seurat v5.1.0. Visium data of LUAD No. 14, which was processed in our previous study[48], was used. (**B**) The expression of the marker genes of alveolar epithelial cells (*HOPX, SFTPA1, SFTPB,* and *SFTPC*), hypoxia (*VEGFA* and *SLC2A1*), and cancer malignancy (*TNC* and *HMGA1*) are shown. The violin plots illustrate the frequency distribution of the expression levels for each Visium spot. The regional pathological and molecular analysis of this specimen using spatial transcriptomic analysis was conducted in our previous study[48]. Tumor tissue of LUAD No.14, which was selected for this study, harbors more heterogeneous histological subtypes compared with other cases. This case includes papillary (moderately-differentiated) and acinar/solid (poorly-differentiated) subtypes. Such heterogeneous lineage differentiation may be associated with aberrant epigenomic changes in the localized regions. Therefore, we used the spatial transcriptomic data obtained by Visium from that study and integrated our new t-nanoEM data with the differential RNA expression in multiple regions.
(**C**) **Number of reads after demultiplexing for lung adenocarcinoma datasets.**
After adapter trimming, the t-nanoEM data, multiplexing five regions dissected from the lung cancer specimen, were demultiplexed using our custom script.
(**D**) **Inter-sample correlation analysis of CpG methylation rates measured by t-nanoEM among dissected samples from the lung adenocarcinoma section.**
CpG methylation rates were compared among t-nanoEM for the microdissected lung cancer tissues. Scatter plots of the methylation rate of CpG covered by five reads or more between short-read EM-seq and t-nanoEM after deduplication. he frequency of the dot counts within each bin is displayed as a heatmap. Pearson's correlation coefficients are shown in each plot.
(**E**) **Expression levels of representative genes in each region on the lung adenocarcinoma section.**
The expression levels of the genes for each region represented in **Figure 5** are shown. The violin plots illustrate the frequency distribution of the expression levels for each Visium spot. Clusters in each region were merged.

Supplementary Reference

S1. Ni, Y., Liu, X., Simeneh, Z.M., Yang, M., and Li, R. (2023). Benchmarking of Nanopore R10.4 and R9.4.1 flow cells in single-cell whole-genome amplification and whole-genome shotgun sequencing. Comput. Struct. Biotechnol. J. *21*, 2352–2364. https://doi.org/10.1016/j.csbj.2023.03.038.

S2. Zou, Z., Ohta, T., and Oki, S. (2024). ChIP-Atlas 3.0: a data-mining suite to explore chromosome architecture together with large-scale regulome data. Nucleic Acids Res. *52*, W45–W53. https://doi.org/10.1093/nar/gkae358.

S3. Weber, S., Eckert, D., Nettersheim, D., Gillis, A.J.M., Schäfer, S., Kuckenberg, P., Ehlermann, J., Werling, U., Biermann, K., Looijenga, L.H.J., et al. (2010). Critical function of AP-2 gamma/TCFAP2C in mouse embryonic germ cell maintenance. Biol. Reprod. *82*, 214–223. https://doi.org/10.1095/biolreprod.109.078717.

S4. Quick, J., Loman, N.J., Duraffour, S., Simpson, J.T., Severi, E., Cowley, L., Bore, J.A., Koundouno, R., Dudas, G., Mikhail, A., et al. (2016). Real-time, portable genome sequencing for Ebola surveillance. Nature 530, 228–232. https://doi.org/10.1038/nature16996.

S5. Leung, A.W.-S., Leung, H.C.-M., Wong, C.-L., Zheng, Z.-X., Lui, W.-W., Luk, H.-M., Lo, I.F.-M., Luo, R., and Lam, T.-W. (2022). ECNano: A cost-effective workflow for target enrichment sequencing and accurate variant calling on 4800 clinically significant genes using a single MinION flowcell. BMC Med. Genomics 15, 43. https://doi.org/10.1186/s12920-022-01190-3.
